# An endoplasmic reticulum-targeting hydroxyl radical fluorescent probe for imaging of ferroptosis and screening of natural protectants†

**DOI:** 10.1039/d4sc07953a

**Published:** 2025-01-28

**Authors:** Hongyu Li, Xue Luo, Yue Jian, Jiajia Lv, Xinmin Li, Jie Gao, Wen Shi, Xiaohua Li, Zeli Yuan, Huimin Ma

**Affiliations:** a College of Pharmacy, Key Laboratory of Basic Pharmacology of Ministry of Education and Joint International Research Laboratory of Ethnomedicine of Ministry of Education, Zunyi Medical University Zunyi Guizhou 563003 China lihongyu@iccas.ac.cn zlyuan@zmu.edu.cn; b Key Laboratory of Analytical Chemistry for Living Biosystems, Institute of Chemistry, Chinese Academy of Sciences Beijing 100190 China mahm@iccas.ac.cn; c Guizhou International Science & Technology Cooperation Base of Medical Optical Theranostics Research Zunyi Guizhou 563003 China; d University of Chinese Academy of Sciences Beijing 100049 China

## Abstract

The real-time and *in situ* detection of hydroxyl radicals (˙OH) in the endoplasmic reticulum (ER) is helpful to understand ferroptosis at its very early stage due to the crucial role of ˙OH in the ER in ferroptosis initiation. Herein, an ER-targeting ˙OH fluorescent probe (ER-OH) has been developed, which showed a large fluorescence increase at 645 nm in response to ˙OH. ER-OH was applied to monitor ferroptosis by fluorescence imaging, revealing a significant increase of the ˙OH level in the ER during this process. With the imaging of ER-OH, a high-throughput screening method was developed to evaluate the anti-ferroptosis activity of a series of natural protectants. Through this screening, the natural flavonoid derivative icariside I was found for the first time to be highly effective in inhibiting ferroptosis by direct scavenging of the excess cytotoxic oxides (*e.g.* ˙OH and lipid peroxides) and restoring the level of GPX4. ER-OH could also be used for *in vivo* imaging of ˙OH in a mouse tumor model. This work provides not only a new tool for ferroptosis monitoring but also a direct insight into the regulation mechanism of ferroptosis and development of new drugs for ferroptosis-related diseases.

## Abstract 2

An endoplasmic reticulum (ER) ·OH fluorescent probe is proposed. Using the probe, the increase of ·OH in the ER during ferroptosis was revealed, and icariside I (a natural flavonoid derivative) was screened for effective inhibition of ferroptosis.

## Graphical abstract

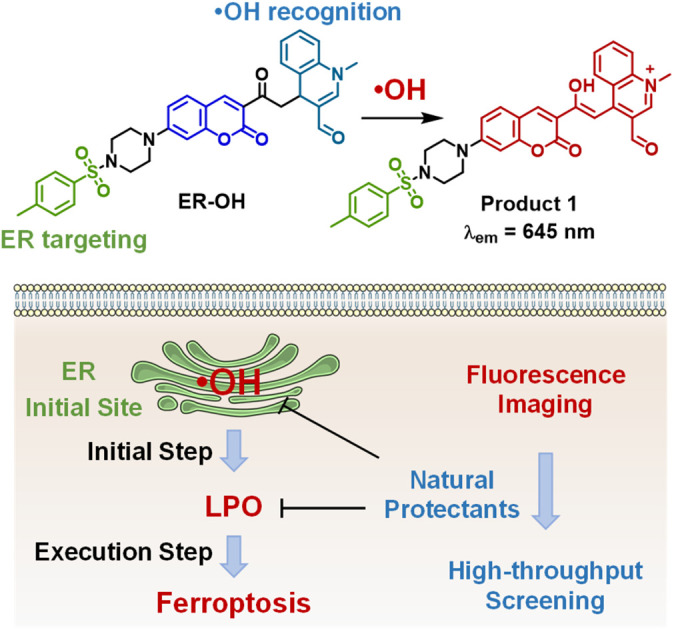

## Introduction

Ferroptosis, an iron-dependent regulated cell death, involves a variety of important physiological and pathological processes, such as senility, immunity, tumorigenesis, ischemia/reperfusion injury, and neurodegenerative diseases.^1–3^ Ferroptosis is driven by lipid peroxidation (LPO) in cellular lipid membranes.^4,5^ The endoplasmic reticulum (ER) is an organelle containing a large lipid membrane structure, comprising about half of the total lipid membrane content in a cell.^6^ It has been reported that the ER is the initial site of LPO accumulation during ferroptosis, which then spreads to other membranes.^7^ Hydroxyl radicals (˙OH) are one of the most important LPO initiators due to their strong oxidation and dehydrogenation ability.^8,9^ In our previous studies, a significant increase in the ˙OH level has been observed during ferroptosis.^10–12^ Therefore, the development of real-time and *in situ* ˙OH assay in the ER may enable monitoring ferroptosis at its very early stage, which is of great significance for the study of ferroptosis and the identification of therapeutic targets for related diseases as well as the screening of new drugs.

Fluorescence imaging has promising application prospects in bioimaging research due to its advantages of high sensitivity, non-invasiveness, real-time imaging and great temporal–spatial sampling capability.^13–17^ A number of fluorescent probes have been proposed for imaging of ˙OH.^18–33^ However, very few of them, including the commercially available ones, can target the ER (Table S1†). Obviously, it is still a great challenge for real-time and *in situ* detection of ˙OH in the ER. This may be due to two reasons. One is the nature of ˙OH, such as high reactivity, short lifetime (nanosecond) and low physiological level (nanomolar),^34^ making it difficult to detect ˙OH in biosystems. The other is that the dense lipid microenvironment of the ER impedes ˙OH capture by the probes. To date, only one ER-targeting ˙OH probe has been reported by our group;^35^ however, its relatively short analytical wavelength (<500 nm) and low sensitivity limit its biological applications. Therefore, there is an urgent need to develop an ER-targeting ˙OH fluorescent probe with higher sensitivity and longer analytical wavelength (>600 nm), which would be beneficial to not only reducing the biological background fluorescence and photodamage but also enhancing the biological tissue penetration of light signals.

**Scheme 1:**
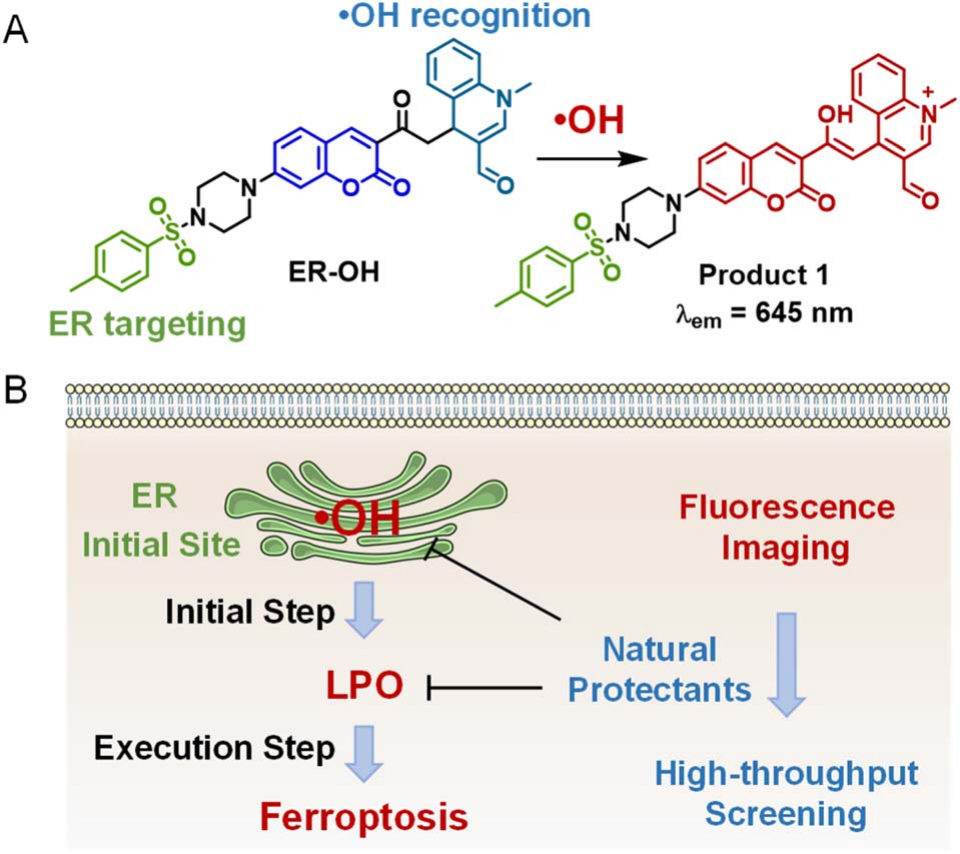
(A) Design and fluorescence response mechanism of ER-OH. (B) The application of ER-OH in imaging of ferroptosis and screening of natural protectants for anti-ferroptosis.

In this work, we report such an ER-targeting ˙OH fluorescent probe ER-OH, which can be used to image ferroptosis. As shown in Scheme 1A, ER-OH is designed based on a stable and bright coumarin fluorophore. In order to increase the sensitivity of the probe, dihydroquinoline, which has specific and sensitive reaction to ˙OH,^26,36^ was selected as the recognition group. Furthermore, an ER-targetable *p*-methyl benzenesulfonamide group was introduced to enable the accumulation of the resulting probe in ER.^37^ After reaction with ˙OH, ER-OH underwent hydrogen abstraction to form the larger π-conjugation and donor–π–acceptor (D–π–A) structure of product 1 (Scheme 1A), which resulted in a large fluorescence increase at 645 nm, thus favoring the sensitive ˙OH detection in the ER. ER-OH has been applied to monitor the ˙OH variations in the ER during ferroptosis. Most importantly, based on the fluorescence imaging of ER-OH and using the initiator of ferroptosis (˙OH in the ER) as a monitoring indicator, a high-throughput screening method has been developed to evaluate the anti-ferroptosis activity of a series of natural protectants (Scheme 1B). Furthermore, the anti-ferroptosis effects of the screened natural protectant icariside I were investigated, revealing its great potential for studying ferroptosis-related diseases.

## Results and discussion

### Analytical properties of ER-OH

**Fig. 1:**
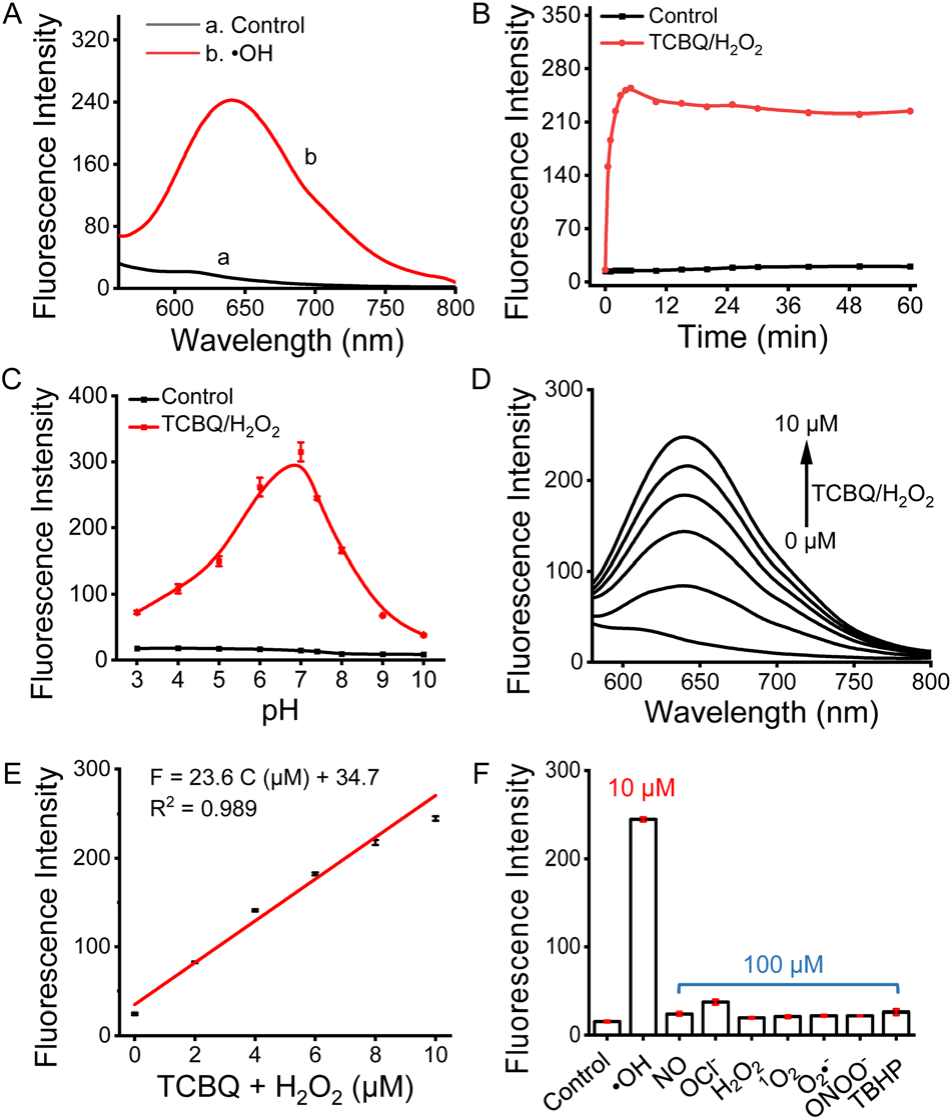
Fluorescence detection of ˙OH in phosphate buffers (20 mM, pH = 7.4) using 10 μM ER-OH. (A) Fluorescence spectra before and after reacting with ˙OH (10 μM TCBQ/H_2_O_2_). (B) Fluorescence response to ˙OH (10 μM TCBQ/H_2_O_2_) with time. (C) Fluorescence response to ˙OH (10 μM TCBQ/H_2_O_2_) at different pH values. (D) Fluorescence spectra with various concentrations of TCBQ/H_2_O_2_. (E) Linear relationship of fluorescence intensity and TCBQ/H_2_O_2_ concentration. (F) Fluorescence responses of ER-OH to ˙OH (10 μM TCBQ/H_2_O_2_) and other common ROS (100 μM). *λ*_ex/em_ = 510/645 nm.

ER-OH and related intermediates were prepared according to the synthetic procedures in Scheme S1 (ESI†) and characterized by ^1^H NMR, ^13^C NMR and HR-ESI-MS (Fig. S1–S13; ESI†). The purity of ER-OH was determined to be 99.1% by HPLC assay (Fig. S14†). The spectroscopic properties of ER-OH in response to ˙OH were investigated in phosphate buffers. ˙OH was generated *via* the *in situ* reaction of tetrachloro-1,4-benzoquinone (TCBQ) and H_2_O_2_.^38^ As shown in Fig. S15,† the maximum absorption of ER-OH is at 420 nm; after reaction with ˙OH, an obvious increase at >500 nm is observed, accompanied by a color change from light yellow to purple (inset of Fig. S15†). On the other hand, ER-OH showed a large fluorescence enhancement of about 20-fold at 645 nm with excitation of 510 nm (Fig. 1A) after the reaction with ˙OH. The distinct spectroscopic changes in absorption and fluorescence implied the formation of product 1 with a larger π-conjugation, which was further confirmed by LC-MS (Fig. S16†) and HR-ESI-MS (Fig. S17†) assays.

Next, the effects of detection conditions, such as pH and reaction time, were investigated. As shown in Fig. 1B and S18,† regardless of light illumination, the fluorescence intensity of ER-OH in the reaction system with TCBQ/H_2_O_2_ reaches a plateau in about 5 min and remains unchanged for at least 60 min; meanwhile, no significant fluorescence change is observed for ER-OH itself. These results suggest the good fluorescence stability of ER-OH. In addition, it was found that ER-OH exhibited significant fluorescence responses to ˙OH under near-physiological pH conditions of 6.0 to 7.4 (Fig. 1C), which may be attributed to the higher ˙OH production efficiency of TCBQ/H_2_O_2_ under near-neutral conditions, consistent with our previous work.^26^ Therefore, a 30 min reaction at physiological pH 7.4 was chosen as the ˙OH detection condition for the subsequent experiments. Furthermore, in the presence of different levels of ˙OH, the fluorescence response of ER-OH showed a linear concentration dependence (Fig. 1D). The linear equation was calculated to be *F* = 23.6 C (μM) + 34.7 (Fig. 1E), with a detection limit (S/N = 3) of 125 nM, which is much lower than 7 μM reported in the literature.^35^ A fast and quantitative fluorescence response was also observed in the detection of ˙OH generated by the Fenton reagent (Fe^2+^-EDTA/H_2_O_2_) but with a stronger response under acidic pH conditions due to the acid dependence of the Fenton reaction (Fig. S19†). Moreover, the detection of ER-OH was not affected by other common reactive oxygen species (ROS; Fig. 1F) and physiologically active species (Fig. S20†).

### Fluorescence imaging performance of ER-OH

**Fig. 2:**
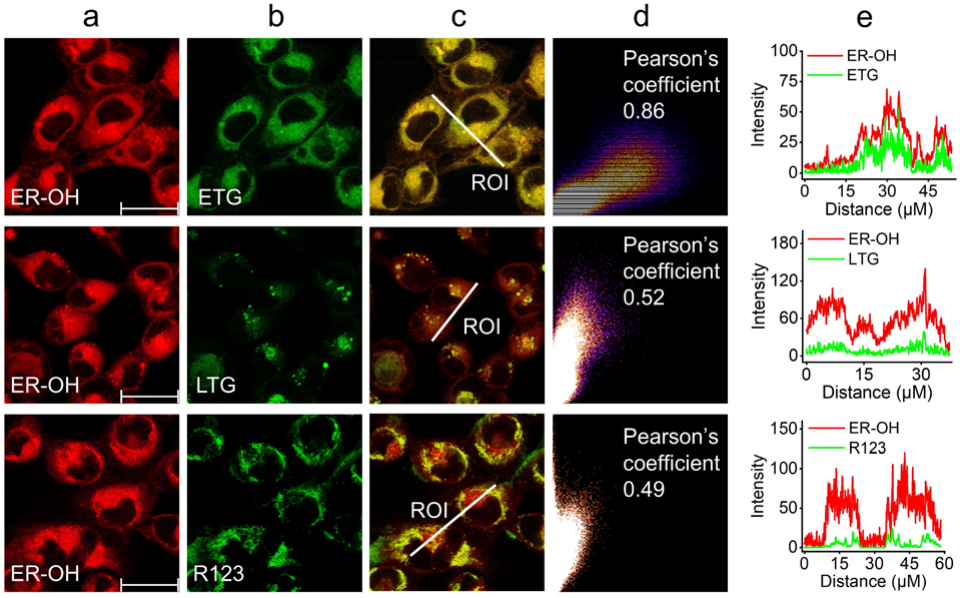
Co-localization imaging of HT-1080 cells co-stained with 10 μM ER-OH and 100 nM organelle dyes and then treated with 100 μM Fenton reagent. (a) ER-OH channel (*λ*_ex_ = 510 nm; *λ*_em_ = 600–700 nm). (b) Organelle dye channel (*λ*_ex_ = 488 nm; *λ*_em_ = 500–560 nm). (c) Merged image of image (a) and image (b). (d) Intensity correlation plot of ER-OH and the organelle dye. (e) Intensity profiles of ER-OH and organelle dye within the white linear region of interest (ROI) in image (c). Scale bars: 30 μm.

The performance of ER-OH for fluorescence imaging of ˙OH in the ER was evaluated. Before that, the cytotoxicity of ER-OH was tested *via* standard MTT assays. It was found that ER-OH showed negligible cytotoxicity to both HeLa and HT-1080 cells at a level of 50 μM after a 24 h incubation (Fig. S21†), suggesting that ER-OH is biocompatible. Then, the ER-targeting ability of ER-OH was studied by colocalization imaging with the commercial organelle dyes, including ER-Tracker Green (ETG), Lyso-Tracker Green (LTG) and Rhodamine 123 (R123). As expected, the fluorescence signal of ER-OH showed a high overlap (Pearson's coefficient: 0.86) with that of ER dye ETG (Fig. 2), in contrast to the poor overlap behaviors with the lysosome dye LTG (Pearson's coefficient: 0.52) and mitochondrial dye R123 (Pearson's coefficient: 0.49), which clearly indicated the favorable ER-targeting ability of ER-OH bearing the *p*-methyl benzenesulfonamide group.

The exogenous ˙OH supplied by the Fenton reagent was then imaged in both HeLa and HT-1080 cells. As shown in Fig. S22,† in the probe-loaded group without exogenous ˙OH (Fig. S22b†), the cells show rather weak fluorescence. However, when the cells were incubated with 50 μM or 100 μM Fenton reagent (Fig. S22c and d†), a concentration-dependent fluorescence increase was produced. Next, to monitor the endogenous production of ˙OH, the cells were pretreated with phorbol-12-myristate-13-acetate (PMA), which could trigger the increase in the intracellular ROS level.^39^ As expected, the PMA treatment (Fig. S23c–e†) could result in significant fluorescence enhancement in both HeLa and HT-1080 cells compared to the untreated cells (Fig. S23b†). In addition, the above increased fluorescence signals produced by exogenous and endogenous ˙OH could be efficiently eliminated upon the addition of the ˙OH scavenger tempol (Fig. S22e and S23f†). These results suggest that ER-OH could be used to monitor the change in the ˙OH level in the ER.

### Monitoring of ER ˙OH variation during ferroptosis

**Fig. 3:**
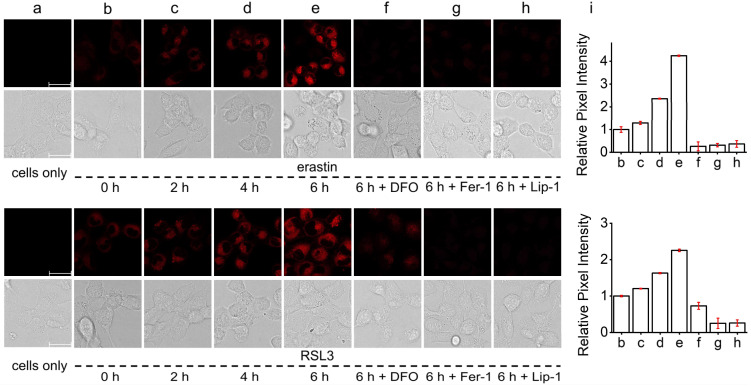
Fluorescence imaging of ER ˙OH in HT-1080 cells during ferroptosis. (a) Cells only. (b–e) Cells pretreated with 10 μM erastin or 2 μM RSL3 for (b) 0 h, (c) 2 h, (d) 4 h or (e) 6 h and then incubated with 10 μM ER-OH for 30 min. (f–h) Cells pretreated with 10 μM erastin or 2 μM RSL3 for 6 h in the presence of (f) 200 μM DFO, (g) 10 μM Fer-1, and (h) 10 μM Lip-1 and then treated with 10 μM ER-OH for 30 min. (i) Relative fluorescence intensities of images (b–h) (fluorescence intensity of image (b) is defined as 1.0). *λ*_ex_ = 510 nm; *λ*_em_ = 600–700 nm. Scar bars, 30 μm.

Because of the favorable ˙OH imaging performance and ER-targeting ability of ER-OH, we next applied it for *in situ* monitoring of ER ˙OH variations during the ferroptosis process. For this, the ferroptosis-susceptible HT-1080 cells were pretreated with two typical ferroptosis initiators with different mechanisms, erastin^40^ and RSL3,^41^ to induce ferroptotic cell death and then stained with ER-OH for fluorescence imaging (Fig. 3). Erastin is a potent inhibitor of the cystine/glutamate antiporter system, can inhibit cystine uptake and lead to the depletion of intracellular biothiols, including glutathione (GSH), which can eliminate lipid peroxides with the catalytic action of the antioxidative glutathione peroxidase 4 (GPX4). As a result, cells treated with erastin would undergo LPO accumulation to the lethal level to induce ferroptosis. Therefore, in the first instance, HT-1080 cells were treated with erastin. It was found that a 2, 4 or 6 h incubation of erastin could lead to a 0.29-, 1.36- or 3.25-fold of fluorescence enhancement, respectively (Fig. 3). On the other hand, RSL3 is a covalent inhibitor of GPX4, which can directly block the elimination of LPO by GPX4 to induce ferroptosis. Similarly, treatment with RSL3 for 2, 4 or 6 h also produced a 0.20-, 0.63- or 1.26-fold fluorescence enhancement, respectively (Fig. 3). These results indicated that both erastin- and RSL3-induced ferroptosis exhibited a significant increase in the ER ˙OH level. More importantly, with the co-incubation of ferroptosis inhibitors, deferoxamine (DFO), liproxstatin-1 (Lip-1) and ferrostatin-1 (Fer-1), the intracellular fluorescence signals of both erastin- and RSL3-treated cells were significantly inhibited. This further established the increase of the ER ˙OH level during ferroptosis and also suggested that ER-OH is a usable tool for monitoring the ˙OH variation in ER.

### High-throughput screening of natural protectants for anti-ferroptosis

**Fig. 4:**
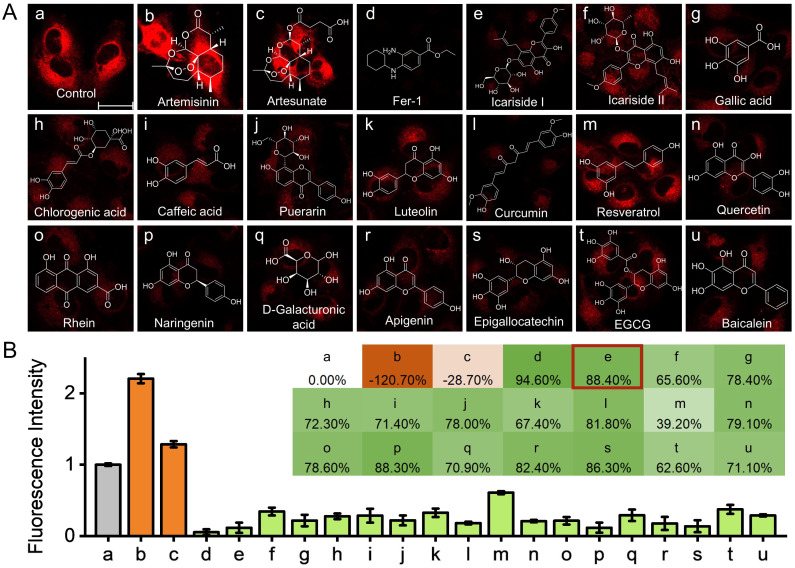
Fluorescence imaging for high-throughput screening of natural protectants for anti-ferroptosis. (A) HT-1080 cells co-treated with 10 μM erastin and 25 μM various natural products for 8 h and then stained with 10 μM ER-OH for 30 min. *λ*_ex_ = 510 nm; *λ*_em_ = 600–700 nm. Scale bar, 30 μm. (B) Relative fluorescence intensities of images (a–u) (fluorescence intensity of image (a) is defined as 1.0). Inset: the ˙OH scavenging efficiency (*E*) of corresponding natural products.

During the ferroptotic cell death, the intracellular antioxidants are unable to remove the excess cytotoxic oxides. Therefore, the introduction of exogenous antioxidants is expected to inhibit ferroptosis.^42^ The commonly used ferroptosis inhibitors (Lip-1 and Fer-1) are typical lipophilic antioxidants.^43^ Small molecule natural products are an important source of new drug development and modification. Natural products from herbs, such as derivatives of flavonoid, caffeic acid and polyphenol, have shown efficient antioxidant effects.^44–47^ Herein, a high-throughput screening method by fluorescence imaging was developed for identifying natural antioxidants with ˙OH scavenging ability and investigating their anti-ferroptosis effects. Based on the advantages of fluorescence imaging technology (*e.g.*, high sensitivity, *in situ* and non-destructive imaging) and taking the initial step of ferroptosis as the monitoring index (that is, ER ˙OH level), it is expected to provide a direct perspective for anti-ferroptosis research. In brief, the cells were co-incubated with the ferroptosis initiator erastin and various natural products for 8 h, respectively, followed by staining with ER-OH for imaging (Fig. 4A). Then the ˙OH scavenging efficiency (*E*) of the corresponding natural products was calculated using the formula *E* (%) = (*F*_1_ − *F*_*x*_)/(*F*_1_ − *F*_0_) × 100%, where *F*_1_, *F*_0_ and *F*_*x*_ represent the fluorescence intensity of ferroptotic cells, normal cells and ferroptotic cells co-incubated with natural products, respectively. As can be seen from Fig. 4, the two reported natural ferroptosis initiators, artemisinin and artesunate^48,49^ (images b and c in Fig. 4A), exhibit a synergistic ferroptosis-promoting effect when co-incubated with erastin, resulting in a significant increase in fluorescence (*i.e.* elevated ER ˙OH level); image d is another control (ferroptosis inhibitor Fer-1). In contrast, all natural antioxidants (images e–u in Fig. 4A) result in a significant decrease in ER ˙OH levels. Among these antioxidants, icariside I, a natural flavonoid derivative from the traditional Chinese herb *Herba Epimedii*, shows the best ˙OH scavenging efficiency of *E* = 88.40% (image e in Fig. 4B). On the other hand, a high ˙OH scavenging efficiency of 80.15% was also found in the RSL3-induced ferroptotic cells co-incubated with icariside I (Fig. S24†). Furthermore, icariside I showed concentration- and time-dependent ˙OH scavenging behavior in both erastin- and RSL3-induced ferroptosis processes (Fig. S25 and S26†). In addition, the ˙OH levels in ferroptotic cells treated with two other ferroptosis inducers, FIN56 and FINO_2_,^50,51^ were monitored by ER-OH imaging, which also revealed the increase of ˙OH levels in these two different ferroptosis pathways (Fig. S27†). Importantly, in the cells treated with FIN56 or FINO_2_, icariside I also showed an efficient ˙OH scavenging ability comparable to that of Fer-1. Taken together, these findings suggested that the elevated ˙OH level is a common feature of the four different ferroptosis pathways; icariside I, as a natural antioxidant, could remove excess intracellular ˙OH and effectively inhibit the ferroptosis process of cells. Icariside I was thus chosen for further studies on anti-ferroptosis effects.

### Studies on anti-ferroptosis effects of icariside I

**Fig. 5:**
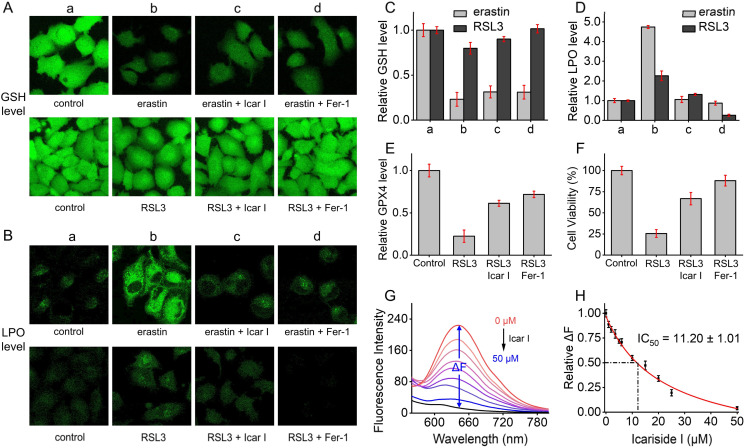
Studies on anti-ferroptosis effects of icariside I (Icar I; 25 μM) in ferroptotic cells induced by 10 μM erastin or 2 μM RSL3. (A) Imaging of the intracellular GSH level using 10 μM monochlorobimane. *λ*_ex_ = 405 nm; *λ*_em_ = 470–520 nm. (B) Imaging of the intracellular LPO level using 10 μM C11-BODIPY 581/591. *λ*_ex_ = 490 nm; *λ*_em_ = 505–550 nm. (C) Relative GSH level after the indicated treatments in panel A. (D) Relative LPO level after the indicated treatments in panel B. (E) Effects of 25 μM icariside I on the GPX4 level of 5 μM RSL3-treated cells. (F) Effects of 25 μM icariside I on the cell viability of 5 μM RSL3-treated cells. (G) Detection of ˙OH (10 μM TCBQ/H_2_O_2_) by 10 μM ER-OH in the presence of icariside I. *λ*_ex_ = 510 nm. Δ*F* represents the difference in fluorescence intensity (*λ*_em_ = 645 nm) after and before the reaction with ˙OH. (H) The IC_50_ value of icariside I for ˙OH scavenging.

To study the anti-ferroptosis effects of icariside I, HT-1080 cells were co-treated with the ferroptosis initiator (erastin or RSL3) and icariside I and then subjected to detecting the key molecular events associated with ferroptosis, such as the levels of GSH, LPO and GPX4. First, the intracellular GSH level was monitored by fluorescence imaging of the commercial GSH probe monochlorobimane. As shown in Fig. 5A and C, erastin, by blocking cystine uptake, causes a significant decrease in intracellular GSH levels, whereas co-incubation with icariside I (as well as the ferroptosis inhibitor Fer-1) does not lead to a recovery of GSH levels; in contrast, RSL3 directly inhibits GPX4 and does not affect intracellular GSH levels, whether icariside I is co-incubated or not. This observation indicated that the anti-ferroptosis activity of icariside I was not mediated by regulation of the intracellular GSH level. Then, the level of LPO (ferroptosis executor) was monitored by C11-BODIPY 581/591. It was found that icariside I could effectively reduce the level of intracellular LPO in both erastin- and RSL3-induced ferroptosis pathways, similar to the well-known lipophilic antioxidant Fer-1 (Fig. 5B and D). Further *in vitro* spectroscopic tests (Fig. S28†) indicated that icariside I is highly effective in scavenging lipid peroxides (produced by lipoxygenase and polyunsaturated fatty acid arachidonic acid), with a half maximal inhibitory concentration (IC_50_) of 8.19 μM. The intracellular level of free or loosely bound Fe^2+^ (labile Fe^2+^) is also a crucial factor of ferroptosis, which was monitored by the commercial Fe^2+^ probe RhoNox-1. Consistent with the previous reports,^52,53^ a significant increase in the Fe^2+^ level was observed in the ferroptotic cells, which could be then suppressed by co-incubation with icariside I or Fer-1 (Fig. S29†). The relative level of the central antioxidative enzyme GPX4 in ferroptosis was also examined (Fig. 5E). After incubation with the GPX4 inhibitor RSL3, the level of GXP4 showed a significant decrease, which, however, was restored with the addition of icariside I. Finally, and most importantly, icariside I demonstrated a potent rescue effect on ferroptotic cell death (Fig. 5F). The direct scavenging effect of icariside I on ˙OH was studied by ER-OH detection (Fig. 5G), revealing an IC_50_ value of 11.20 μM (Fig. 5H), comparable to that of lipid peroxides. Taken together, these results suggest that icariside I, as a natural antioxidant, can inhibit ferroptosis by direct scavenging of the excess cytotoxic oxides (˙OH and lipid peroxides) and restoring the level of GPX4 during ferroptosis, indicating its great potential in the treatment of ferroptosis-related diseases.

### *In vivo* imaging of ˙OH

The feasibility of ER-OH for *in vivo* fluorescence imaging was further explored in a tumor model, which was reported to be accompanied by a significant increase in the ROS level.^54^ 5 week old SPF BALB/c female mice were selected to establish an ectopic 4T1 tumor model for imaging, and all animal experiments were approved by the Experimental Animal Ethics Committee of Zunyi Medical University (approval number: [2021]2-422). As shown in Fig. S30,† after *in situ* injection of ER-OH, only very weak fluorescence is observed in the normal tissues (on the left dorsum); however, the tumor tissue (on the right dorsum) shows strong fluorescence, which can be maintained for a long imaging time (more than 48 h). This result also demonstrates the ability of ER-OH for *in vivo* imaging, enabling its application in a wider range of physiological and pathological conditions.

## Conclusions

In conclusion, ER-OH as an ER-targeting ˙OH fluorescent probe has been developed for imaging ferroptosis in this work. ER-OH was designed based on a coumarin fluorophore, a dihydroquinoline recognition group, and an ER-targetable *p*-methyl benzenesulfonamide group. ER-OH showed a significant fluorescence increase at 645 nm in response to ˙OH due to the formation of the larger π-conjugation and D–π–A structure. ER-OH has been applied to monitor ferroptosis, revealing the increase of the ER ˙OH level during this process. Moreover, based on the fluorescence imaging of ER-OH, a high-throughput screening method was developed to evaluate the anti-ferroptosis activity of a series of natural protectants. Through this screening, the natural flavonoid derivative icariside I has been found for the first time to be highly effective in inhibiting ferroptosis by direct scavenging of the excess cytotoxic oxides and restoring the level of GPX4. ER-OH could also be used for *in vivo* imaging of ˙OH in the mouse tumor model. These studies provide not only a new tool for ferroptosis monitoring but also a direct insight into the regulation mechanism of ferroptosis and development of new drugs for ferroptosis-related diseases.

## Author contributions

Hongyu Li: methodology, validation, investigation, writing – original draft. Xue Luo: investigation, visualization. Yue Jian: investigation. Jiajia Lv: investigation. Xinmin Li: investigation. Jie Gao: investigation. Wen Shi: investigation. Xiaohua Li: investigation. Zeli Yuan: supervision, writing – review & editing. Huimin Ma: supervision, writing – review & editing.

## Conflicts of interest

There are no conflicts to declare.

## Supplementary Material

SC-016-D4SC07953A-s001

## Data Availability

Supplementary data for this article, including materials and instruments, experimental methods, synthesis and characterization, and supplementary figures, are provided in the ESI.†
