# Supplementary material for: An endoplasmic reticulum-targeting hydroxyl radical fluorescent probe for imaging of ferroptosis and screening of natural protectants
Source: Chem Sci. 2025 Jan 28;16(9):4136–43. doi: 10.1039/d4sc07953a (PMC11788824; doi:10.1039/d4sc07953a)
Supplement: SC-016-D4SC07953A-s001 [file SC-016-D4SC07953A-s001.pdf]

Supplementary Information (SI) for Chemical Science.
This journal is © The Royal Society of Chemistry 2025

Electronic Supplementary Information for

An Endoplasmic Reticulum-targeting Hydroxyl Radical Fluorescence Probe for Imaging of Ferroptosis and Screening of Natural Protectants

Hongyu Li,*[ac] Xue Luo,[ac] Yue Jian,[ac] Jiajia Lv,[ac] Xinmin Li,[ac] Jie Gao,[ac] Wen Shi,[bd] Xiaohua Li,[b] Zeli Yuan,*[ac] Huimin Ma*[bd]

[a] *College of Pharmacy, Key Laboratory of Basic Pharmacology of Ministry of Education and Joint International Research Laboratory of Ethnomedicine of Ministry of Education, Zunyi Medical University, Zunyi, Guizhou 563003, China.*

[b] *Key Laboratory of Analytical Chemistry for Living Biosystems, Institute of Chemistry, Chinese Academy of Sciences, Beijing 100190, China.*

[c] *Guizhou International Science & Technology Cooperation Base of Medical Optical Theranostics Research, Zunyi, Guizhou 563003, China.*

[d] *University of Chinese Academy of Sciences, Beijing 100049, China.*

Table of contents

Materials and instruments....2

Synthesis and characterization of ER-OH....2

Methods....11

Detection of •OH....14

Biocompatibility of ER-OH....16

Imaging of exogenous and endogenous •OH in living cells....16

Imaging of the effect of icariside I on ferroptosis....17

Fluorescence imaging of mice....20

Comparison of ER-OH with existing •OH probes....21

References....24

Materials and instruments

1-Boc piperazine, 4-fluorosalicylaldehyde, ethyl acetoacetate, rhodamine 123 and phorbol-12-myristate-13-acetate (PMA) were obtained from Energy Chemical. Erastin, RSL3, ferrostatin-1 (Fer-1) and liproxstatin-1 (Lip-1) were obtained from TargetMol. Tempol and deferoxamine mesylate (DFO) were obtained from Sigma-Aldrich. ER-Tracker Green and Lyso-Tracker Green were obtained from Beyotime biotechnology Co., LTD (Shanghai, China). The standard substances of natural products were obtained from Desite biotechnology Co. LTD (Chengdu, China). Monochlorobimane and C11-BODIPY 581/591 were obtained from GlpBio. Human glutathione peroxidase 4 (GPX4) ELISA kit was purchased from Ruixin biotechnology Co., LTD (Quanzhou, China).

NMR spectra were measured on a DD2 400 MHz spectrometer (Agilent, USA). Absorption spectra and fluorescence spectra were measured by a UV 2600i spectrophotometer (Shimadzu, Japan) and a Cary Eclipse spectrophotometer (Varian, USA), respectively. Fluorescence imaging of living cells was performed by a STELLARIS 5 confocal laser scanning microscope (Leica, Germany) and image processing were conducted by ImageJ software. Fluorescence imaging of mice was performed by a Nightowl LB 983 imaging system (Berthold, Germany).

Synthesis and characterization of ER-OH

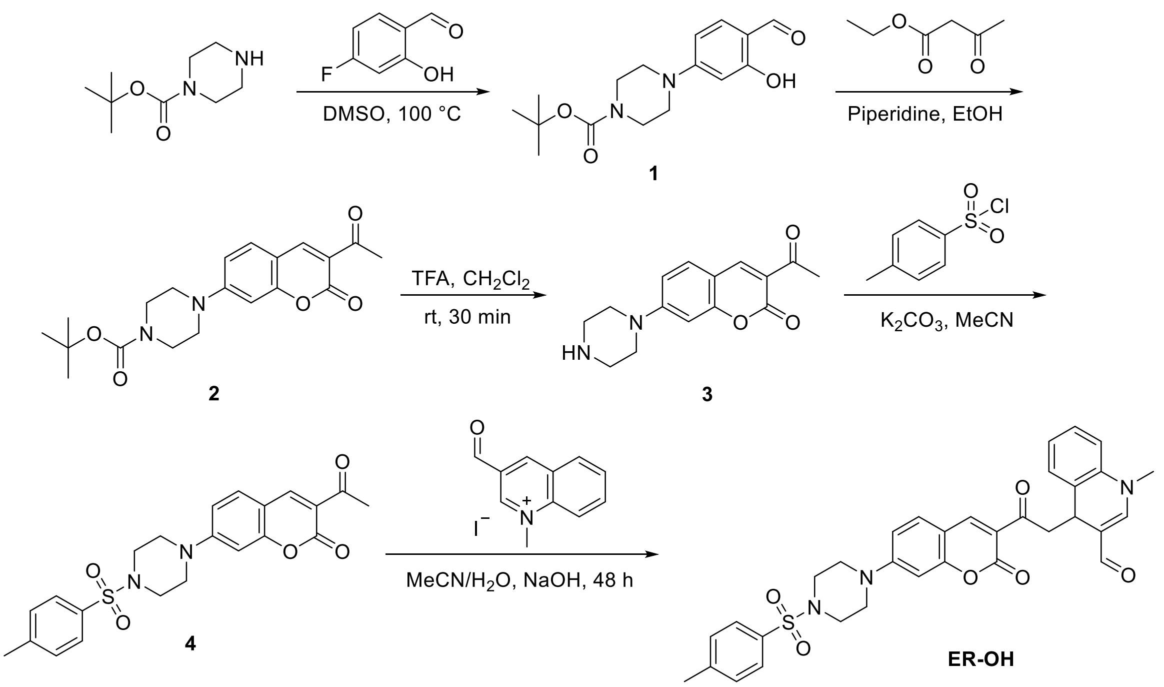

Scheme S1 Synthetic procedures of ER-OH.

*Synthesis of compound **1***

1-Boc piperazine (2.23 g, 12 mmol) and 4-fluorosalicylaldehyde (1.40 g, 10 mmol) were dissolved in 25 mL of DMSO, then heated to 100 °C for 22 h. After that, 50 mL of deionized water was added. The mixture was then extracted by ethyl acetate (20 mL × 3). The organic phase was washed with saturated brine, and dried with anhydrous Mg_2SO_4, then removed the solvent under reduced pressure. The residue was purified by silica gel column chromatography (petroleum ether : ethyl acetate = 2 : 1, v/v) to obtain compound **1** as yellow solid (2.30 g, 75% yield). ^{1}H NMR (400 MHz, DMSO-d_6): δ 11.15 (s, 1H), 9.74 (s, 1H), 7.49 (d, J = 8.9 Hz, 1H), 6.57 (dd, J = 9.1, 2.1 Hz, 1H), 6.30 (d, J = 2.3 Hz, 1H), 3.46 – 3.38 (m, 8H), 1.42 (s, 9H). ^{13}C NMR (101 MHz, DMSO-d_6): δ 191.6, 163.5, 156.4, 154.2, 133.7, 113.3, 106.7, 99.2, 79.6, 46.3, 46.2, 28.5.

*Synthesis of compound **2***

Compound **1** (3.06 g, 10 mmol) and ethyl acetoacetate (1.69 g, 13 mmol) were dissolved in 200 mL anhydrous ethanol and 2 mL piperidine. The mixture was heated to reflux 4 h, then cooled down in an ice water bath to form orange-yellow precipitates. The precipitates were filtrated and dried to obtain compound **2** (2.95 g, yield 79.4 %). ^{1}H NMR (400 MHz, DMSO-d_6): δ 8.54 (s, 1H), 7.73 (d, J = 9.0 Hz, 1H), 7.02 (d, J = 9.0 Hz, 1H), 6.85 (d, J = 2.3 Hz, 1H), 3.51 (d, J = 6.5 Hz, 4H), 3.46 (d, J = 6.0 Hz, 4H), 2.52 (s, 3H), 1.42 (s, 9H). ^{13}C NMR (101 MHz, DMSO-d_6): δ 194.9, 159.9, 158.2, 155.3, 154.2, 148.1, 132.6, 117.3, 112.0, 109.3, 98.7, 79.6, 56.5, 46.4, 30.7, 28.5.

*Synthesis of compound **3***

Compound **2** (3.72 g, 10 mmol) was dissolved in 70 mL CH_2Cl_2 and 30 mL trifluoroacetic acid. The solution was stirred for 30 min at room temperature, then the solvent was removed under reduced pressure. The residue was purified by silica gel column chromatography (CH_2Cl_2 : MeOH = 10 : 1, v/v) to afford compound **3** as yellow solid (2.14 g, 78.2 % yield). ^{1}H NMR (400 MHz, Chloroform-d): δ 8.44 (s, 1H), 7.44 (d, J = 8.9 Hz, 1H), 6.82 (dd, J = 8.9, 2.4 Hz, 1H), 6.65 (d, J = 2.5 Hz, 1H), 3.45 – 3.40 (m, 4H), 3.05 – 3.01 (m, 4H), 2.69 (s, 3H), 1.82 (s, 1H). ^{13}C NMR (101 MHz, Chloroform-d): δ 195.8, 160.6, 158.4, 155.7, 147.9, 131.7, 118.0, 111.7, 109.7, 99.2, 48.1, 45.8, 30.8. HR-ESI-MS: *m/z* calcd for compound **3** ($C_{15}H_{16}N_2O_3Na^+$, $[M + Na]^+$), 295.1053; found, 295.1061.

Synthesis of compound ***4***

Compound **3** (2.72 g, 10 mmol), *p*-toluenesulfonyl chloride (2.86 g, 15 mmol) and K_2CO_3 (2.76 g, 20 mmol) were dissolved 40 mL acetonitrile, then heated at 40 °C for 8 h. After cooling down to room temperature, the solvent was removed under reduced pressure. The solid residue was then purified by silica gel column chromatography (CH_2Cl_2 : MeOH = 100 : 1, v/v) to obtain compound **4** as yellow solid (2.03 g, 47.5 % yield). ^{1}H NMR (400 MHz, Acetonitrile-d_3): δ 8.43 (s, 2H), 7.62 (s, 1H), 7.59 (s, 1H), 6.95 (d, J = 2.4 Hz, 1H), 6.92 (d, J = 2.4 Hz, 1H), 6.75 (d, J = 2.3 Hz, 2H), 3.79-3.76 (m, 4H), 3.60-3.57 (m, 4H), 2.57 (s, 3H), 2.17 (s, 3H). ^{13}C NMR (101 MHz, Acetonitrile-d_3): δ 195.9, 161.4, 159.0, 155.7, 148.2, 132.8, 131.0, 129.7, 129.1, 119.6, 116.1, 112.6, 110.7, 100.1, 47.8, 46.6, 43.5, 30.5. HR-ESI-MS: *m/z* calcd for compound **4** ($C_{22}H_{22}N_2O_5SNa^+$, $[M + Na]^+$), 449.1142; found, 449.1145.

Synthesis of compound ER-OH

Compound **4** (853 mg, 2 mmol), 3-formyl-1-methylquinolin-1-ium iodide [1] (413 mg, 2.4 mmol) and NaOH (0.40 mg, 10 mmol) were dissolved in 40 mL ethanol and H_2O (1:1 v/v). The reaction was stirred at 35 °C for 24 h in dark, and removed solvent under reduced pressure. The residue was purified by silica gel column chromatography (CH_2Cl_2 : MeOH = 100 : 1) to obtain ER-OH as yellow powder (148 mg, 12.4%). ^{1}H NMR (400 MHz, Chloroform-d): δ 9.10 (s, 1H), 8.35 (s, 1H), 7.64 (d, J = 8.1 Hz, 2H), 7.46 (d, J = 6.0 Hz, 1H), 7.39 (d, J = 8.9 Hz, 1H), 7.33 (d, J = 8.0 Hz, 2H), 7.17 (t, J = 6.9 Hz, 1H), 7.05 (t, J = 7.4 Hz, 1H), 6.98 (s, 1H), 6.86 (d, J = 8.2 Hz, 1H), 6.71 (dd, J = 8.9, 2.4 Hz, 1H), 6.55 (d, J = 2.4 Hz, 1H), 4.70 (t, J = 6.1 Hz, 1H), 3.59 (dd, J = 15.3, 5.7 Hz, 1H), 3.49 (t, J = 5.1 Hz, 4H), 3.40 (s, 3H), 3.12 (d, J = 5.0 Hz, 4H), 3.00 (dd, J = 15.3, 6.6 Hz, 1H), 2.41 (s, 3H). ^{13}C NMR (101 MHz, Chloroform-d): δ 193.7, 186.9, 160.0, 157.8, 154.3, 151.8, 147.8, 144.2, 138.1, 133.3, 132.0, 131.6, 130.0, 129.9, 127.8, 126.9, 124.6, 119.0, 114.8, 112.8, 111. 9, 110. 5, 100.0, 52.1, 46.7, 44.9, 39.4, 30.8, 21.6. HR-ESI-MS: *m/z* calcd for ER-OH ($C_{33}H_{31}N_3O_6SNa^+$, $[M + Na]^+$), 620.1826; found, 620.1830.

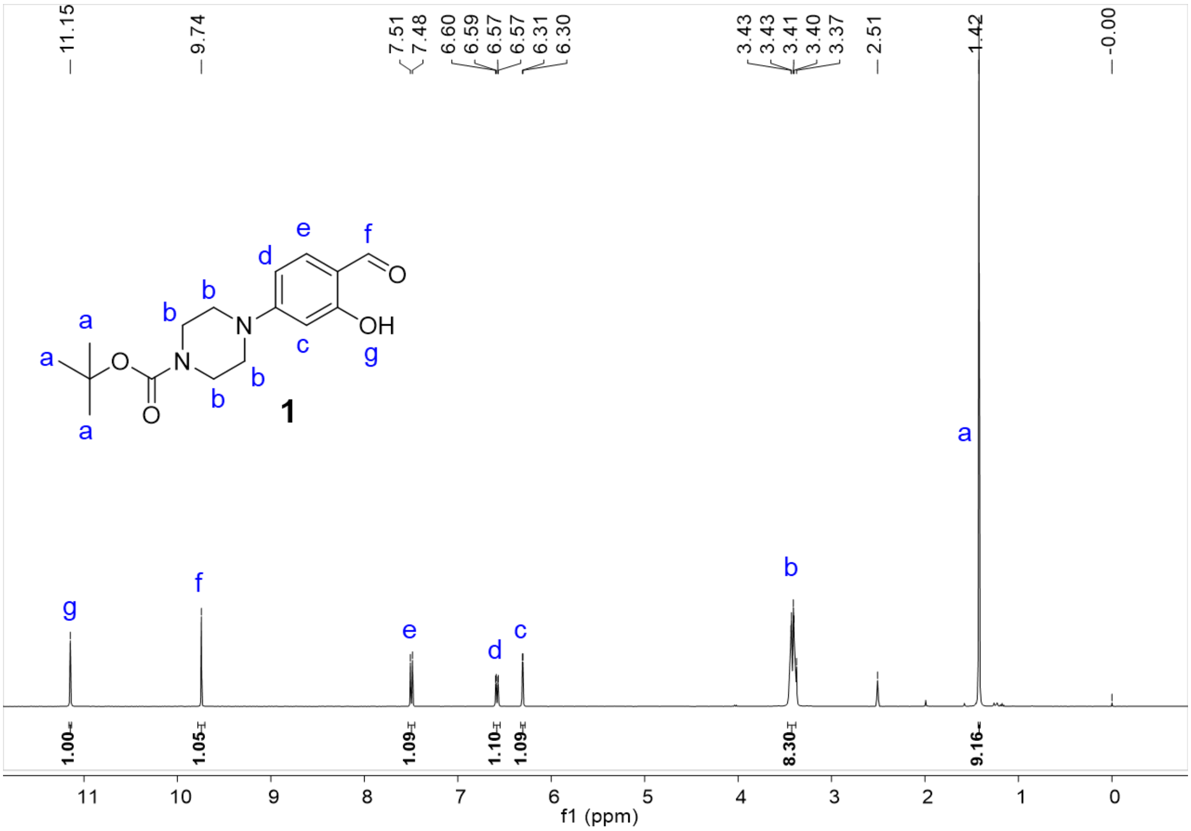

Fig. S1 ^{1}H NMR spectrum of compound **1** in DMSO-d_6 (400 MHz, 298 K).

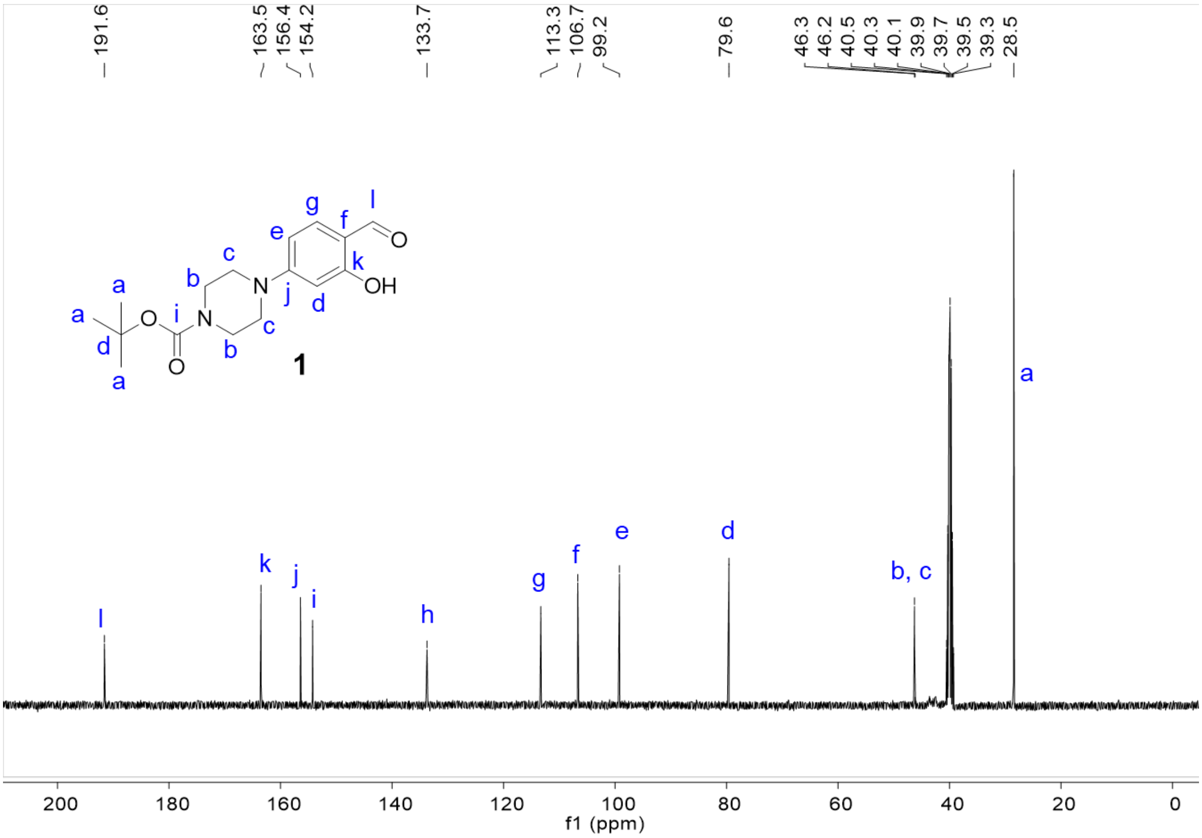

Fig. S2 ^{13}C NMR spectrum of compound **1** in DMSO-d_6 (101 MHz, 298 K).

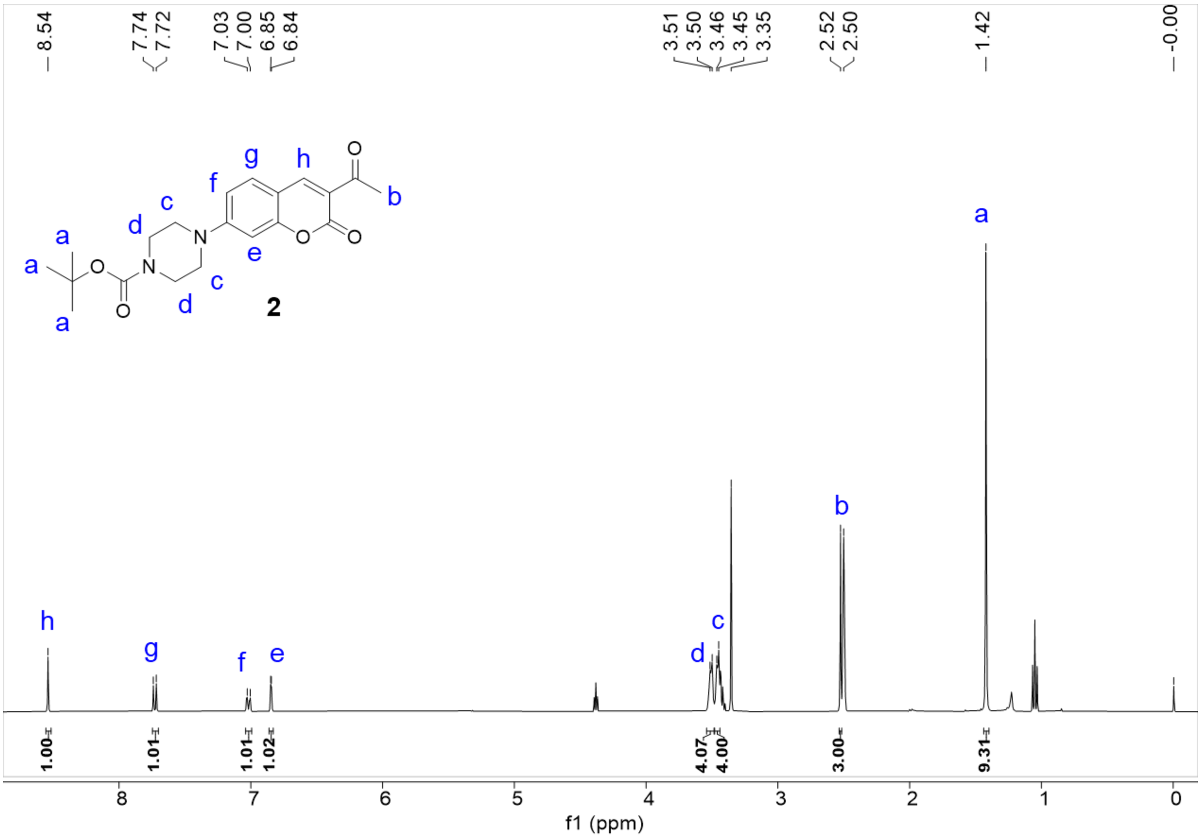

Fig. S3 ^{1}H NMR spectrum of compound **2** in DMSO-d_6 (400 MHz, 298 K).

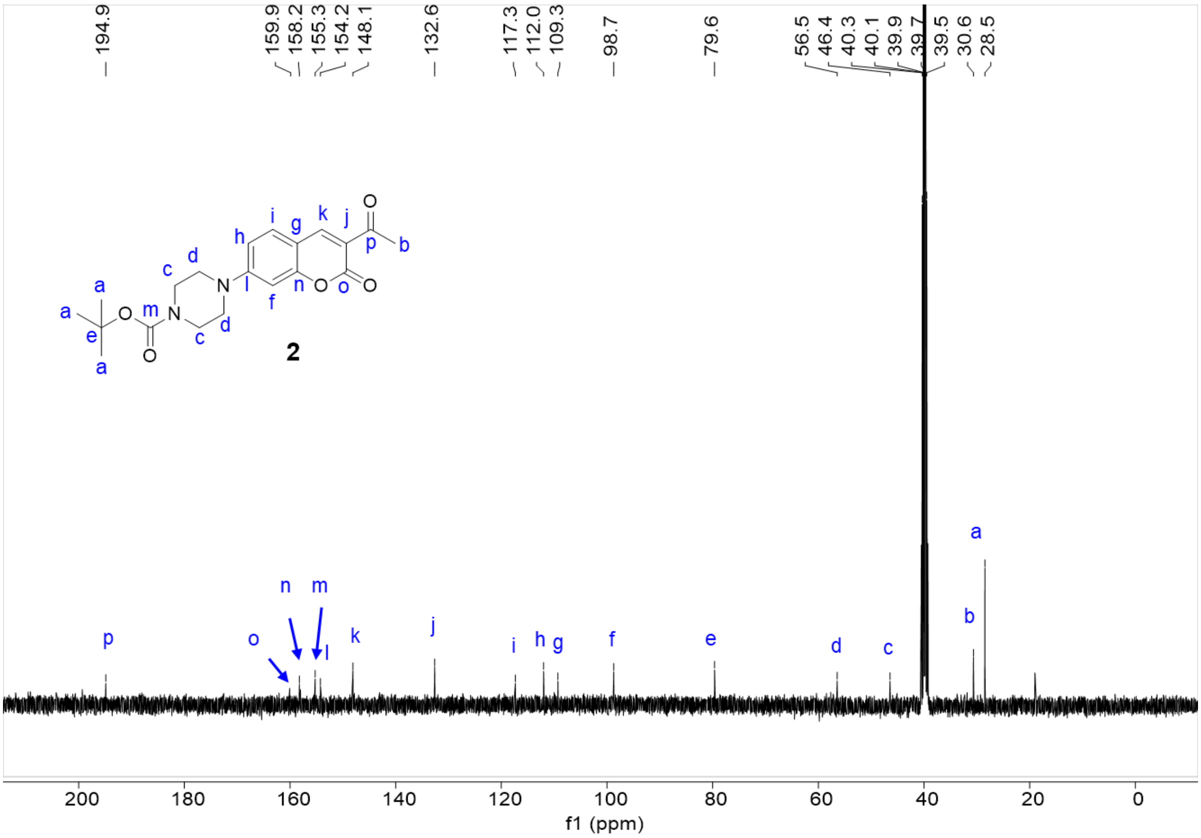

Fig. S4 ^{13}C NMR spectrum of compound **2** in DMSO-d_6 (101 MHz, 298 K).

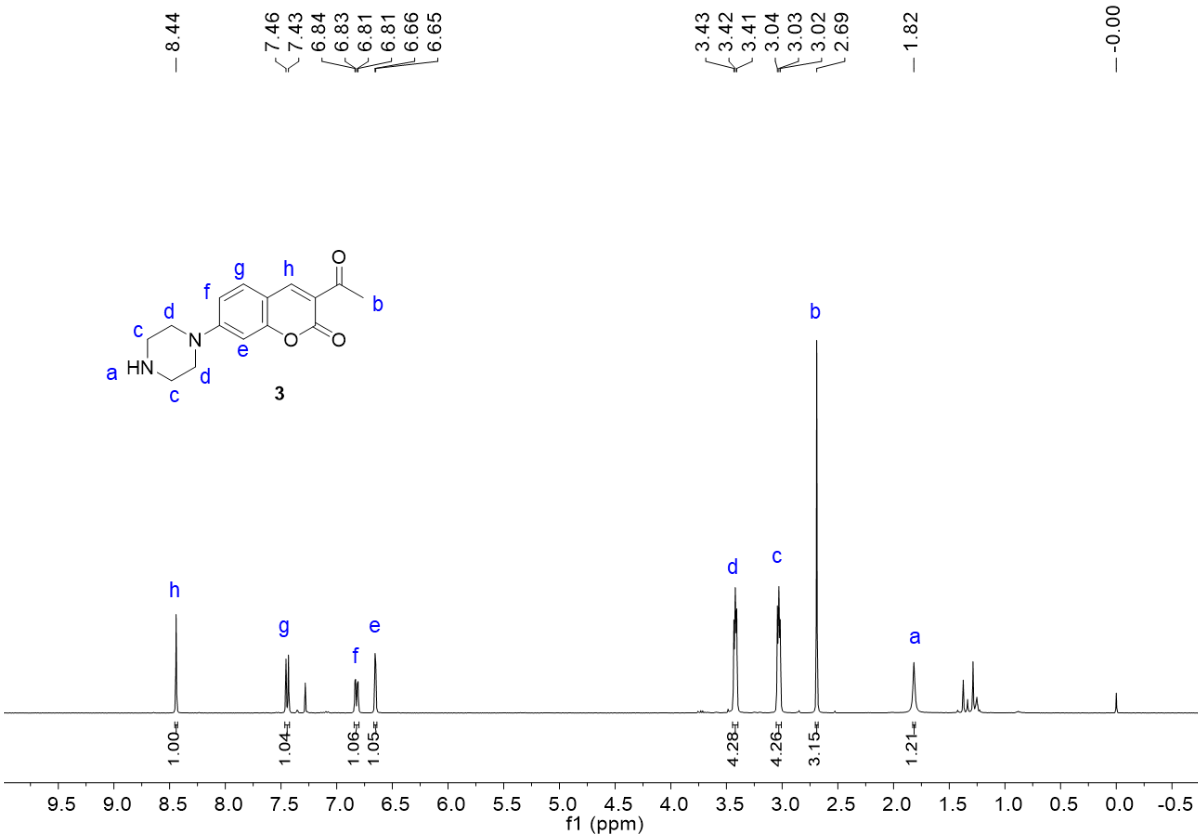

Fig. S5. ^{1}H NMR spectrum of compound **3** in $CDCl_3$ (400 MHz, 298 K).

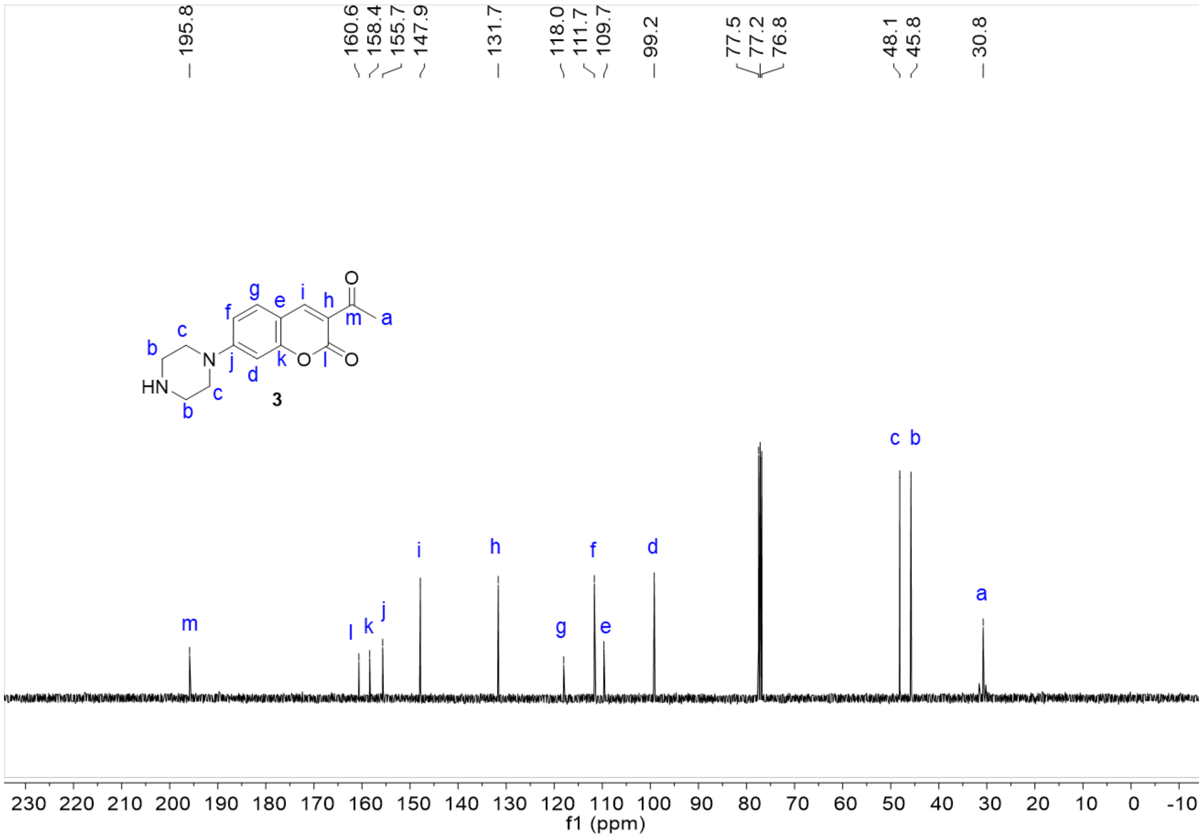

Fig. S6 ^{13}C NMR spectrum of compound **3** in $CDCl_3$ (101 MHz, 298 K).

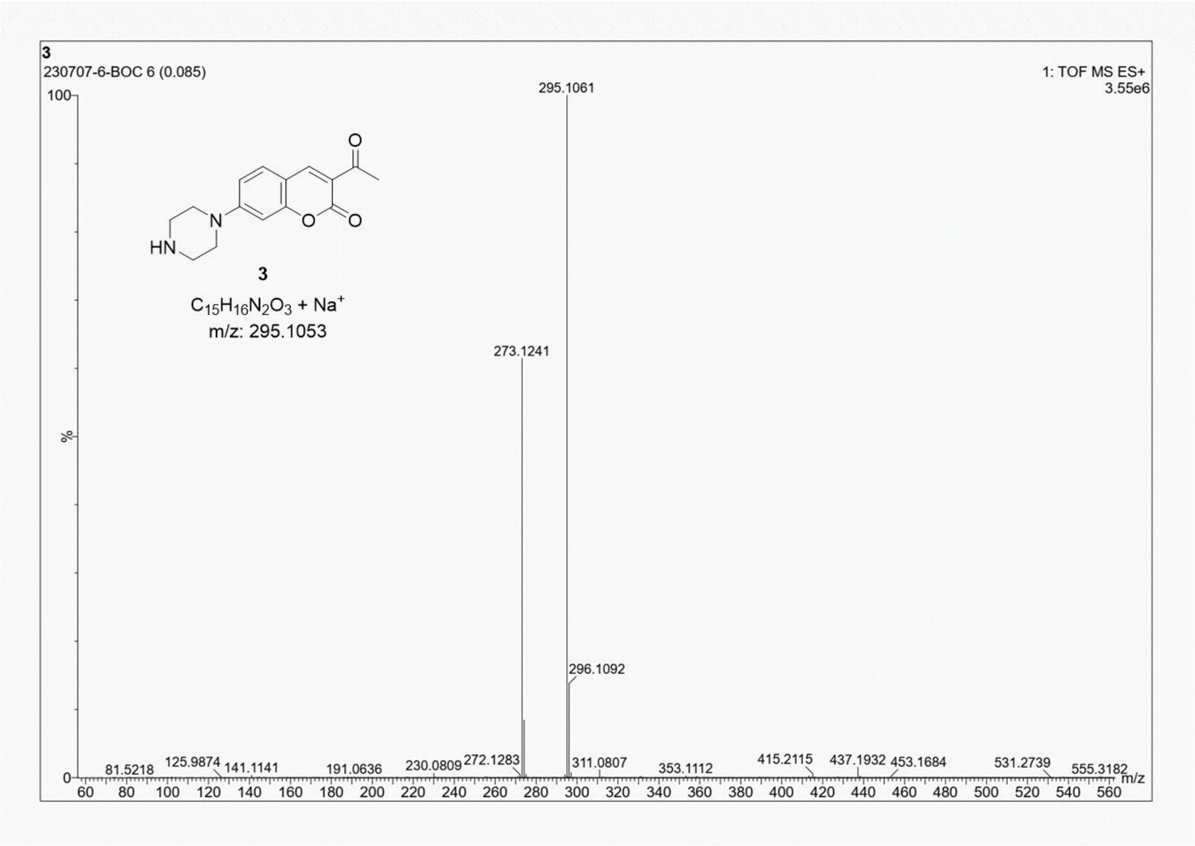

Fig. S7 HR-ESI-MS spectrum of compound **3**.

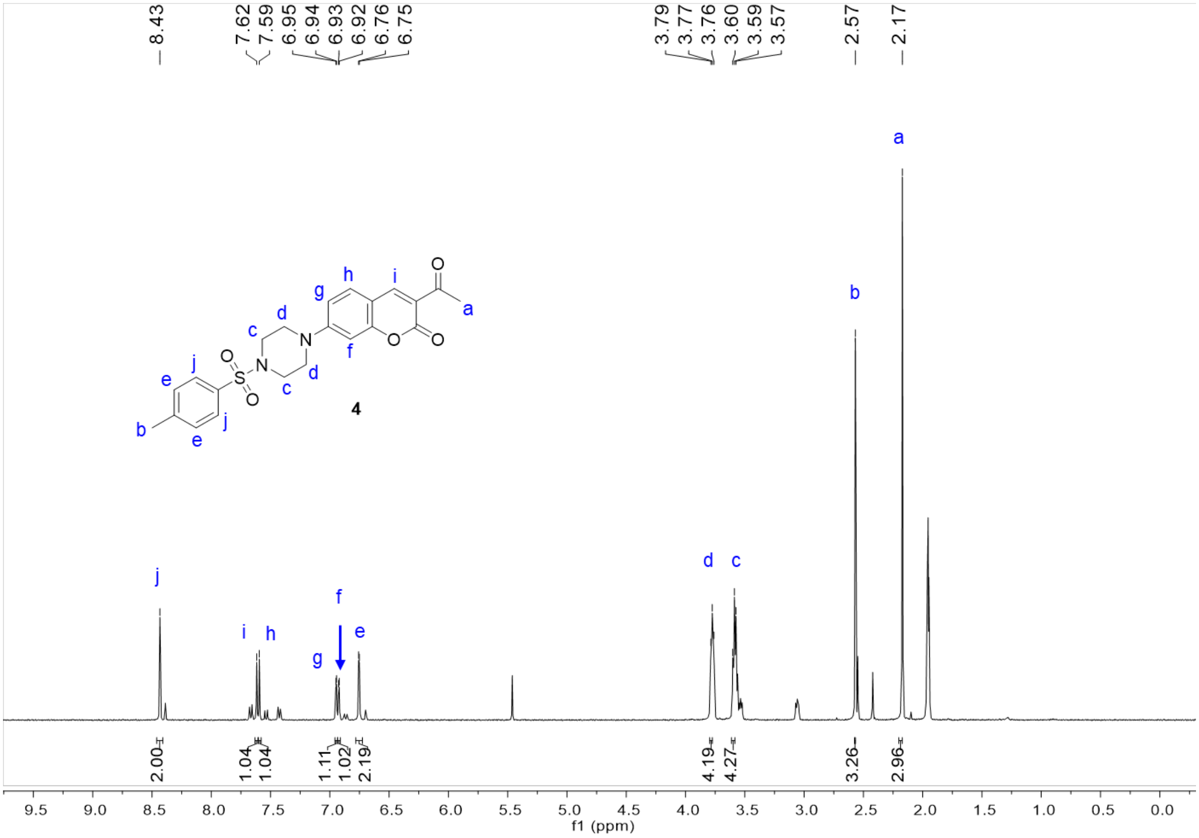

Fig. S8 ^{1}H NMR spectrum of compound **4** in Acetonitrile-d_3 (400 MHz, 298 K).

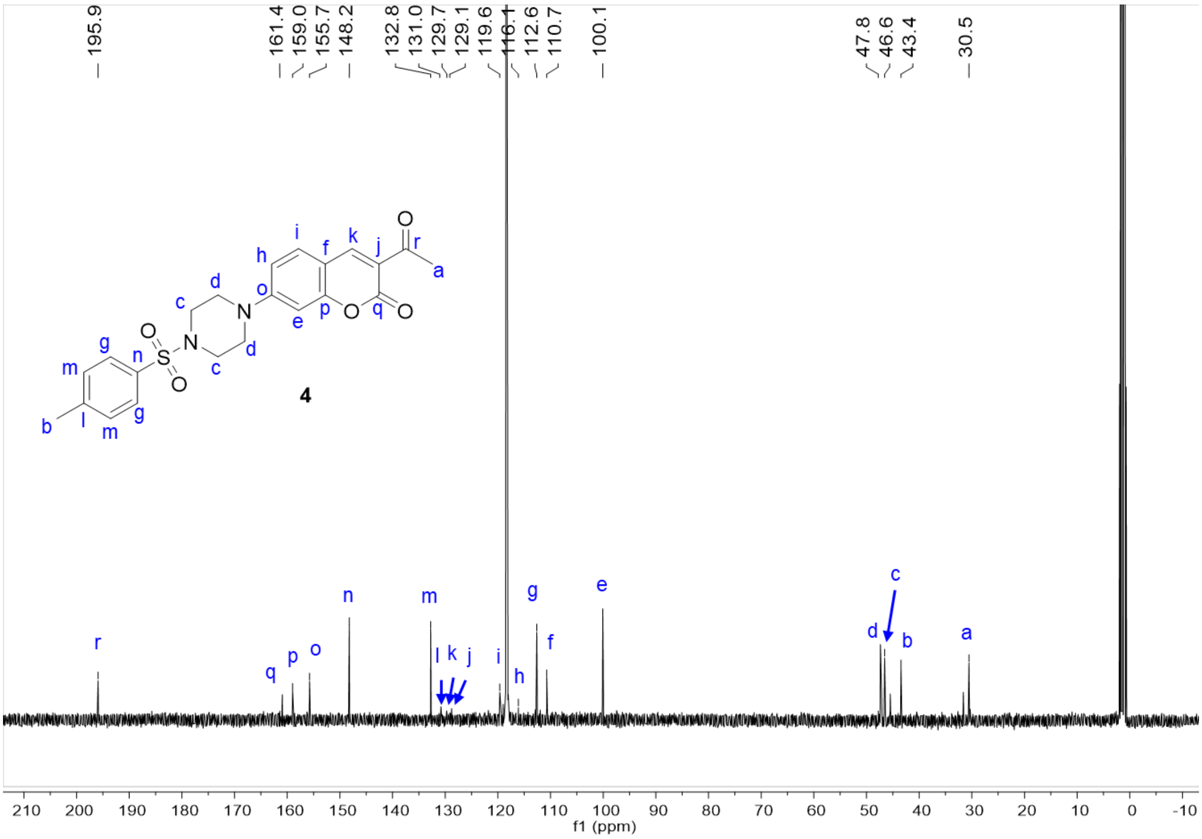

Fig. S9 ^{13}C NMR spectrum of compound **4** in Acetonitrile-d_3 (101 MHz, 298 K).

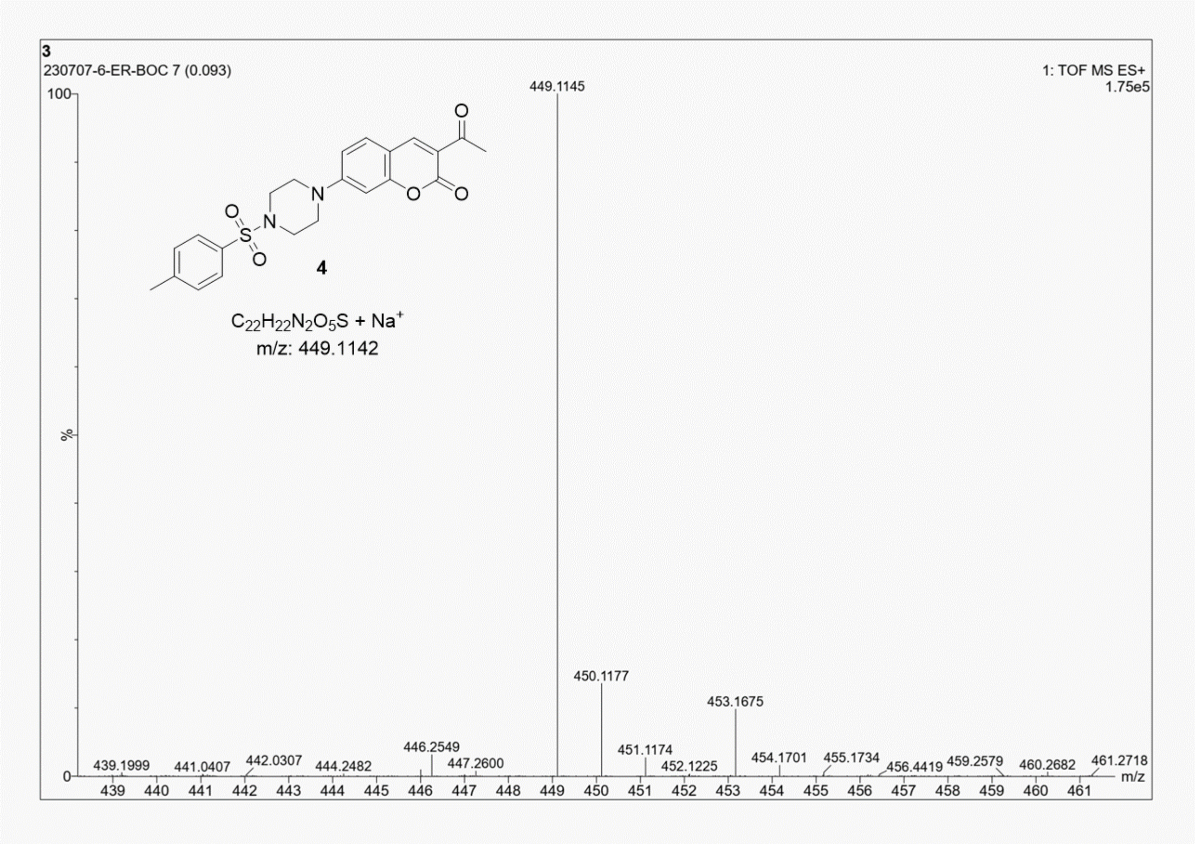

Fig. S10 HR-ESI-MS spectrum of compound **4**.

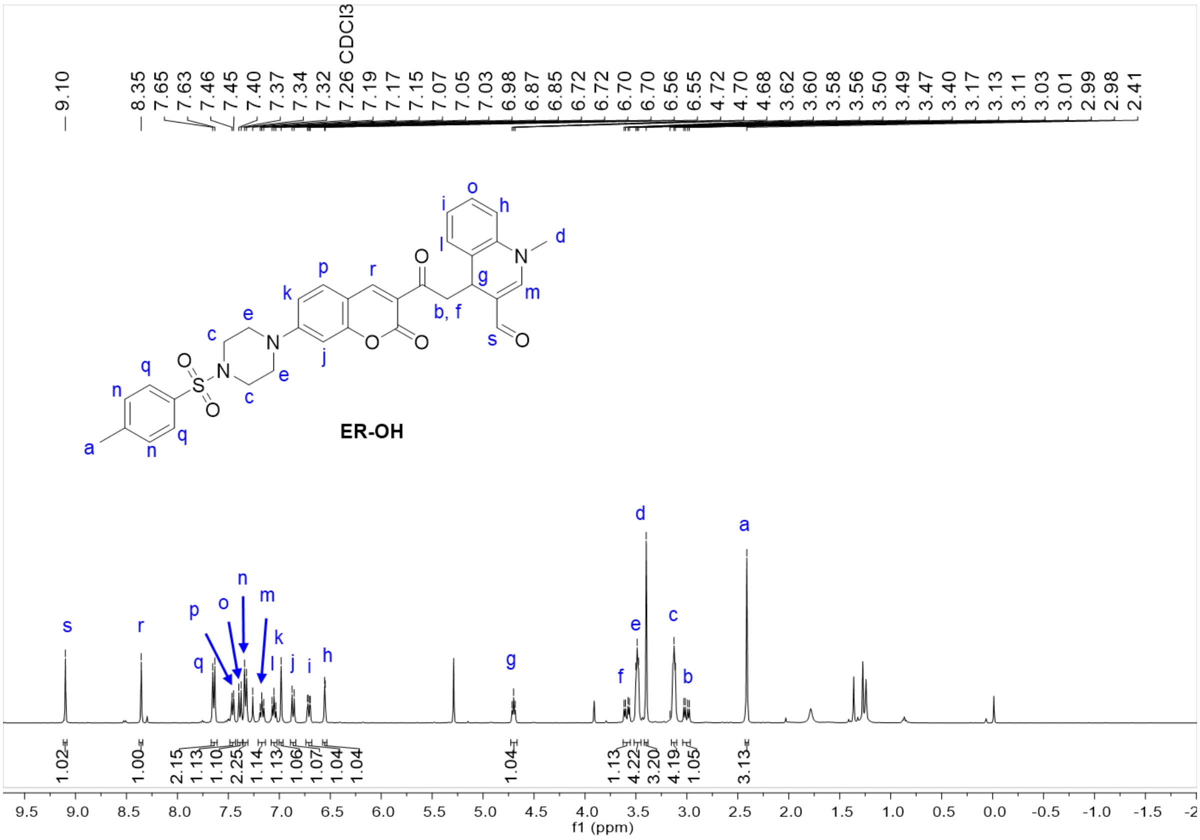

Fig. S11 ^{1}H NMR spectrum of ER-OH in $CDCl_3$ (400 MHz, 298 K).

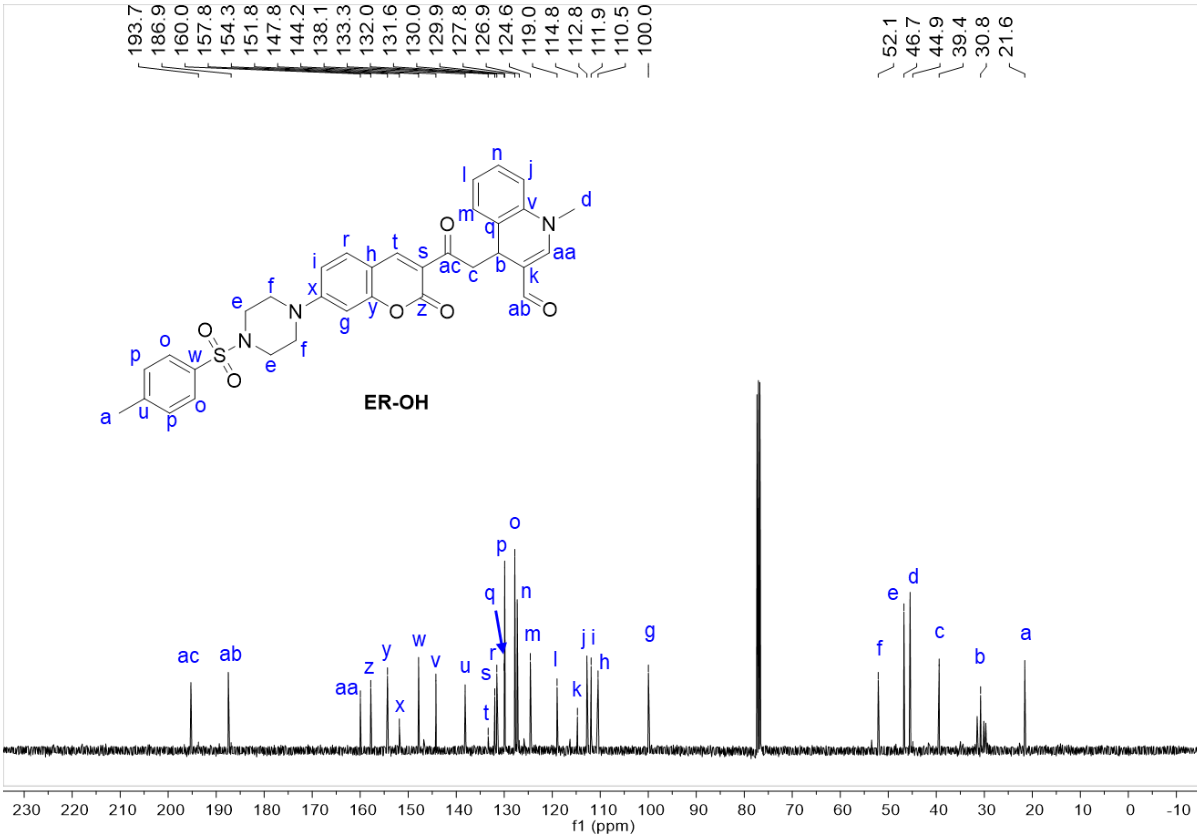

Fig. S12 ^{13}C NMR spectrum of ER-OH in $CDCl_3$ (101 MHz, 298 K).

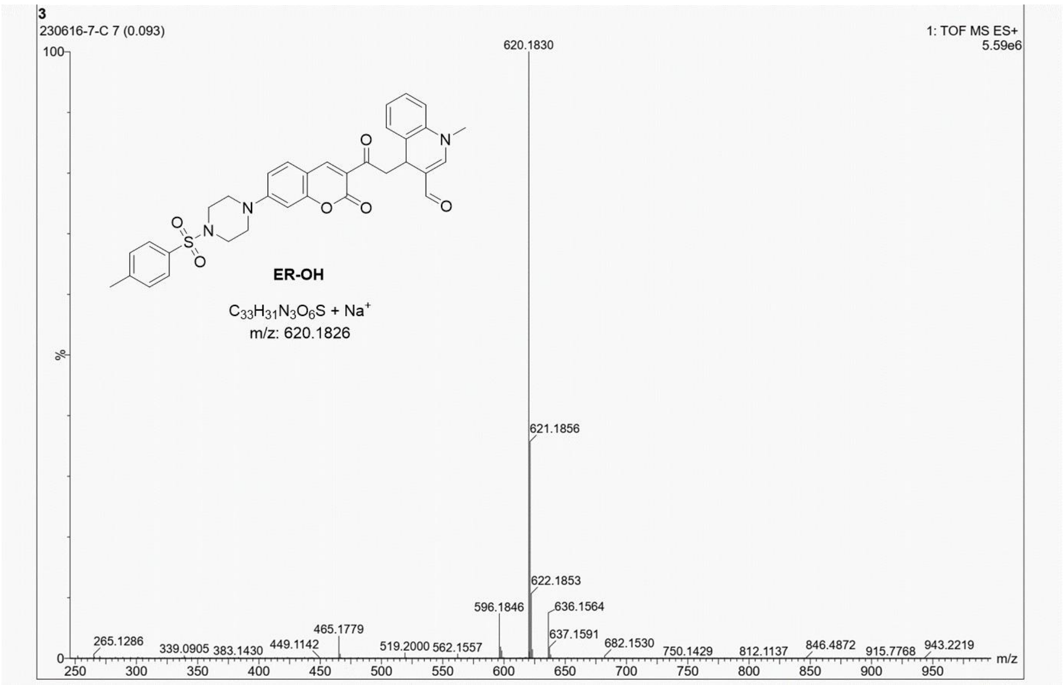

Fig. S13 HR-ESI-MS spectrum of ER-OH.

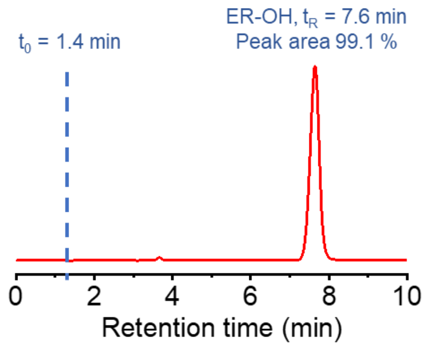

Fig. S14 HPLC assay of ER-OH. The purity of ER-OH was determined to be 99.1% (peak area).

Methods

General procedures for spectroscopic measurements

Unless otherwise mentioned, all spectral measurements for •OH were performed in 20 mM phosphate buffers as follow: to the test tubes, 3 mL of phosphate buffer and 10 μL of ER-OH stock solution (1 mM in DMF; final concentration 10 μM) was added, and followed by an appropriate amount of reactant (TCBQ/H_2O_2, Fe^{2+}-EDTA/H_2O_2 or other analytes). The resulting mixtures were allowed to react for 30 min left at room temperature and then subjected to spectroscopic measurements. Unless otherwise stated, the results were presented as mean ± standard deviation (n = 3).

Cell culture

HT-1080 cells were cultured in RPMI 1640 medium containing 10% (v/v) new-born bovine serum, 100 U/mL penicillin, and 100 μg/mL streptomycin. HeLa cells and 4T1 cells were cultured in DMEM medium containing 10% fetal bovine serum, 100 U/mL penicillin, and 100 μg/ mL streptomycin. All the cells were cultured at 37°C in a humidity of 95% air and 5% CO_2. After growing to approximately 70% confluence, the cells were treated with trypsin, and then re-cultured in the glass-bottom dishes for 24 h for the next experiments.

Fluorescence imaging of •OH in cells

For fluorescence imaging for exogenous •OH in living cells, HT-1080 or HeLa cells were incubated with 10 μM ER-OH for 30 min. Then, the cells were washed three times with serum-free medium, and incubated with 50 μM or 100 μM Fenton reagents (Fe^{2+}-EDTA and H_2O_2) for 30 min for fluorescence imaging. For scavenging of •OH, 100 μM tempol (•OH scavenger) was added and co-incubated with 100 μM Fenton reagents. Fluorescence imaging was performed on a confocal laser scanning microscope with a 510 nm excitation source by collecting fluorescence signal from 600 nm to 700 nm (similarly hereinafter).

For fluorescence imaging of endogenous •OH in living cells, HT-1080 or HeLa cells were incubated with 2 μg/mL PMA for 2, 4, and 6 h, respectively. Then, the cells were washed three times with serum-free medium, and treated with 10 μM ER-OH for 30 min for imaging. For scavenging of •OH, the PMA-treated (6 h) cells were added with 100 μM tempol for 30 min before staining with ER-OH.

For fluorescence imaging of •OH in ferroptosis process, HT-1080 cells were treated with 10 μM erastin or 2 μM RSL3 for 2, 4 and 6 h to induce ferroptosis. After removal of the medium, the cells were washed three times with serum-free medium, and then incubated with 10 μM ER-OH for 30 min for •OH imaging. In the ferroptosis inhibition group, the cells were incubated with 10 μM erastin or 2 μM RSL3 in the presence of 200 μM DFO, 10 μM Fer-1 or 10 μM Lip-1.

High-throughput screening of natural products

HT-1080 cells were co-treated with 10 μM erastin (or 2 μM RSL3) and 25 μM various natural products for 8 h, respectively. Then, the culture medium was removed, and the cells were washed three times with serum-free medium, and finally stained with 10 μM ER-OH for

30 min for fluorescence imaging. The •OH scavenging efficiency (E) of the corresponding natural products were calculated as formula S1.

$$E\ (\%) = (F_1\text{-}F_x)/(F_1\text{-}F_0) \times 100\% \qquad \text{(S1)}$$

where F_1 is the fluorescence intensity of ferroptotic cells, F_0 is the fluorescence intensity of normal cells, and F_x is the fluorescence intensity of ferroptotic cells co-incubated with natural products.

Studies on the anti-ferroptosis effects of icariside I

HT-1080 cells were divided into four groups and treated as follow: (1) without any treatment; (2) 10 μM erastin (or 2 μM RSL3) for 8 h; (3) 10 μM erastin (or 2 μM RSL3) + 25 μM icariside I for 8 h; (4) 10 μM erastin (or 2 μM RSL3) + 25 μM Fer-1 for 8 h. After treatment, all the above cells were washed by serum-free medium for three times for the following experiments. For cell viability assay, the cells were subjected to standard MTT assays.[2] The GPX4 levels of the cells were detected by GPX4 ELISA kit according to the manufacturer's protocols. For fluorescence imaging of GSH and lipid peroxidation levels, the cells were treated with 10 μM monochlorobimane or C11-BODIPY 581/591 for 30 min. For monochlorobimane, fluorescence imaging was performed with an excitation of 405 nm, and fluorescence signals were collected in the range of 470 nm-520 nm; for C11-BODIPY 581/591, fluorescence imaging was performed with an excitation of 490 nm, and fluorescence signals were collected in the range of 505 nm-550 nm.

Imaging of •OH in tumor models of mice:

5-week-old SPF BALB/c female mice were selected to establish an ectopic 4T1 tumor model. All animal experiments were approved by the Experimental Animal Ethics Committee of Zunyi Medical university (approval number: [2021]2-422). The mice were acclimatized for one week at 25°C and 40 - 60% humidity. Then, the right dorsum of the mice was subcutaneously injected with 100 μL of 4T1 cells (1×10^7 cells/mL) to establish ectopic 4T1 tumor models, which was allowed to grow to about 100 mm^3. Fluorescence imaging was performed after *in situ* injection of 20 μL ER-OH (1 mM in pH 7.4 PBS containing 10% DMF) into the tumor tissue, as well as the normal tissue on the left dorsum as a control.

Detection of •OH

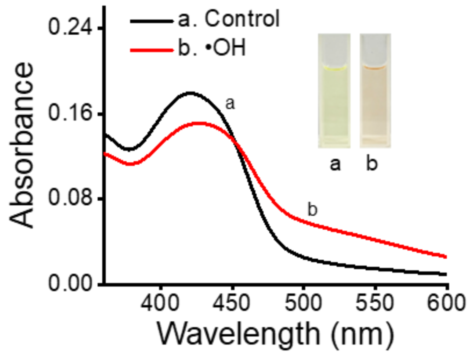

Fig. S15 Absorption spectra of ER-OH (10 μM) (a) before and (b) after reacting with •OH (10 μM TCBQ/H_2O_2).

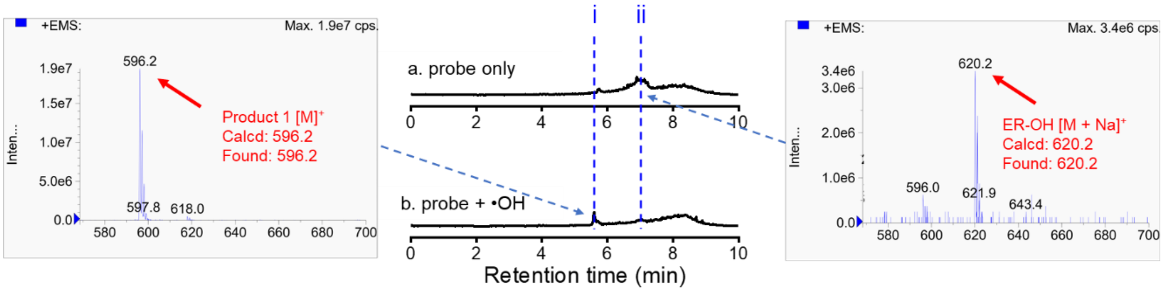

Fig. S16 LC-MS assays of different reaction systems: (a) ER-OH only; b) ER-OH reacting with •OH (TCBQ + H_2O_2) in acetonitrile. The assignments of the peaks: (i) t_R = 5.6 min, product 1 (m/z = 596.2); (ii) t_R = 7.0 min, ER-OH (m/z = 620.2).

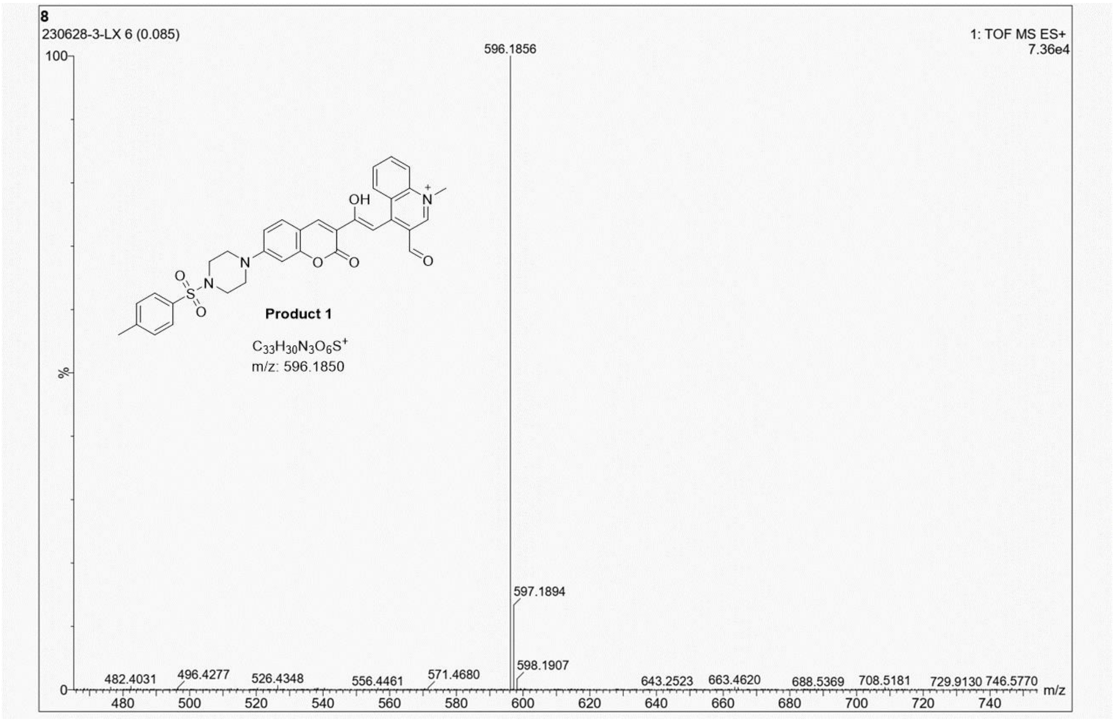

Fig. S17 HR-ESI-MS spectrum of ER-OH after reacting with •OH.

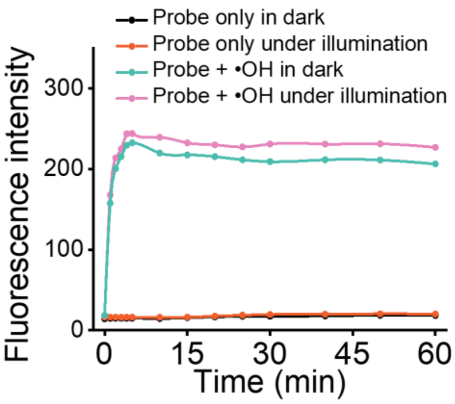

Fig. S18 Fluorescence intensity of 10 μM Lyso-OH itself or its reaction with •OH (10 μM TCBQ + 10 μM H_2O_2) under a white light illumination (0.4 W/cm^2) or in the dark. $\lambda_{ex/em}$ = 510/645 nm.

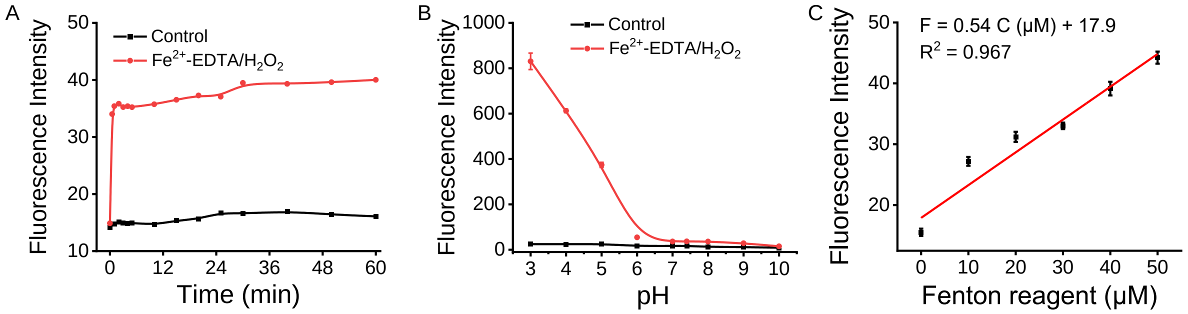

Fig. S19 Effects of (A) reaction time and (B) pH on the fluorescence response of ER-OH (10 μM) to •OH from Fenton reagent (50 μM Fe^{2+}-EDTA/ H_2O_2). (C) Linear relationship of fluorescence intensity and Fenton reagent concentration. $\lambda_{ex/em}$ = 510/645 nm.

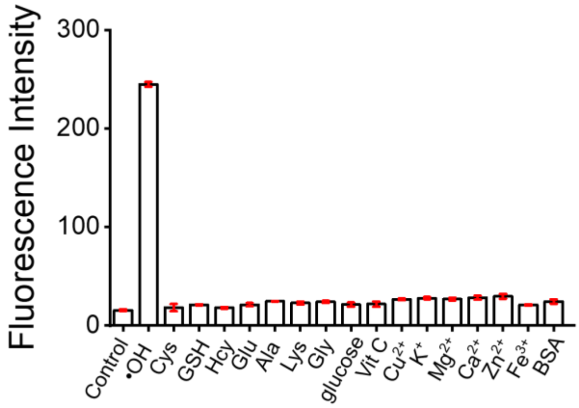

Fig. S20 Fluorescence responses of 10 μM ER-OH toward various common bioactive species and metal ions in pH 7.4 phosphate buffer: •OH (20 μM TCBQ + 20 μM H_2O_2); 1 mM Cys; 5 mM GSH; 100 μM Hcy; 1 mM Glu; 1 mM Ala; 1 mM Lys; 1 mM Gly; 10 mM glucose; 1mM Vit C; 100 μM Cu^{2+}; 150 mM K^+; 2 mM Mg^{2+}; 100 μM Ca^{2+}; 100 μM Zn^{2+}; 100 μM Fe^{3+}; 30 μg/mL BSA. $\lambda_{ex/em}$ = 510/640 nm.

Biocompatibility of ER-OH

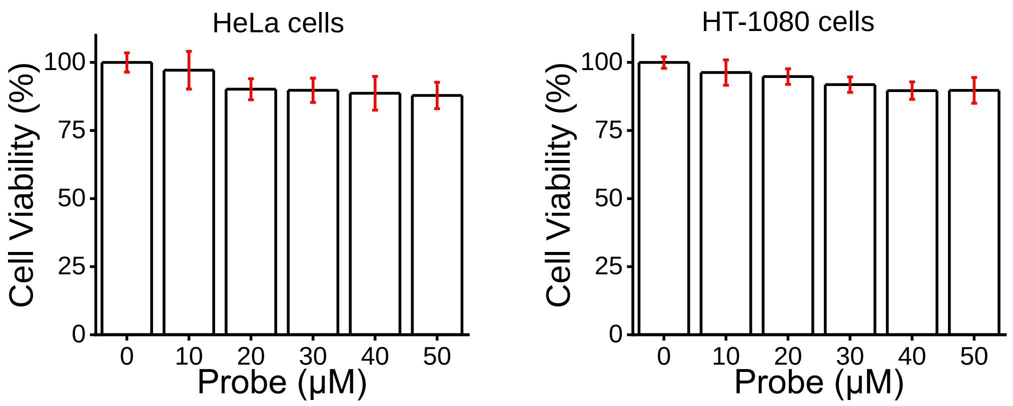

Fig. S21 The viability of different cells after treated with various amounts of ER-OH for 24 h. The viability of the untreated cells (0 μM) is defined as 100%. The results are presented as mean ± standard deviation (n = 6).

Imaging of exogenous and endogenous •OH in living cells

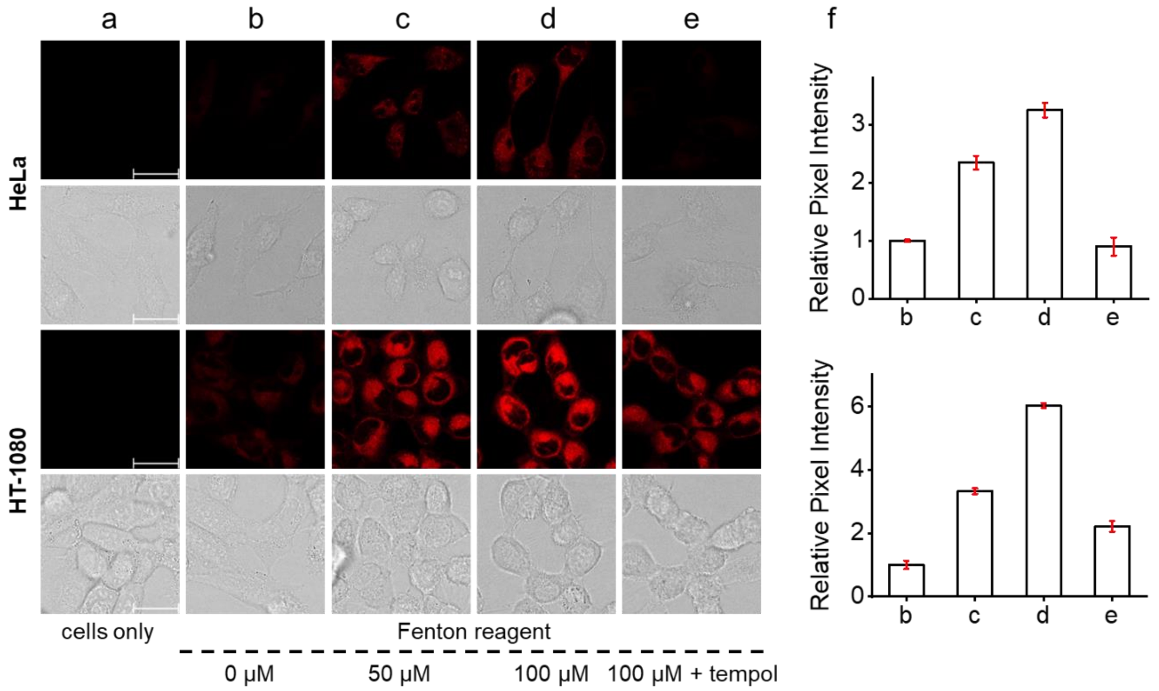

Fig. S22 Fluorescence imaging of •OH in HeLa and HT-1080 cells treated by Fenton reagent (Fe^{2+}-EDTA + H_2O_2). (a) Cells only. (b-e) Cells incubated with 10 μM ER-OH for 30 min, then with (b) 0 μM, (c) 50 μM, (d) 100 μM Fenton reagent or (e) 100 μM Fenton reagent and 200 μM tempol for 30 min. λ_{ex} = 510 nm, λ_{em} = 600–700 nm. Scale bars, 30 μm. (f) Relative pixel intensity of images b-e of HeLa and HT-1080 cells. Note: the pixel intensity of the corresponding image b is defined as 1.0; the results are presented as mean ± standard deviation (n = 3).

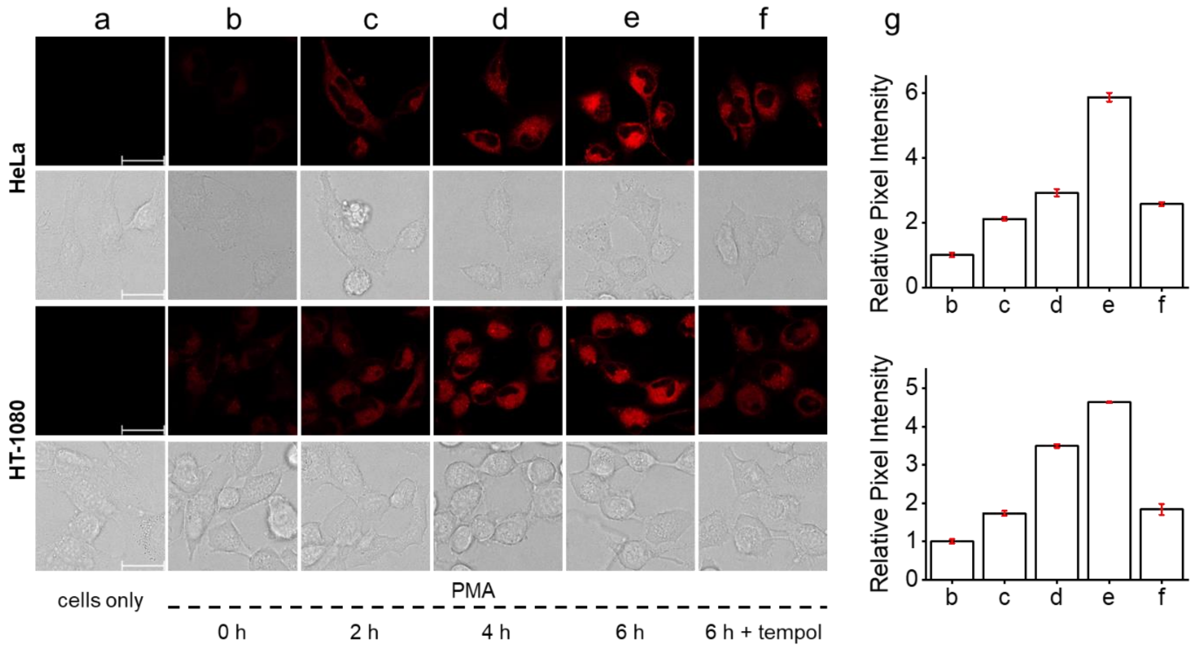

Fig. S23 Fluorescence imaging of •OH in HeLa and HT-1080 cells stimulated by PMA. (a) Cells only. (b-e) Cells pretreated with PMA (10 µg/mL) for (b) 0 h, (c) 2 h, (d) 4 h or (e) 6 h, and then incubated with ER-OH (10 µM) for 30 min. (f) Cells pretreated with PMA (10 µg/mL) for 6 h, then incubated with tempol (200 µM) for 30 min, and finally stained with ER-OH (10 µM) for 30 min. λ_{ex} = 510 nm, λ_{em}= 600-700 nm. Scale bars, 30 µm. (g) Relative pixel intensity of images b-f of HeLa and HT-1080 cells. Note: the pixel intensity of the corresponding image b is defined as 1.0; the results are presented as mean ± standard deviation (n = 3).

Imaging of the effect of icariside I on ferroptosis

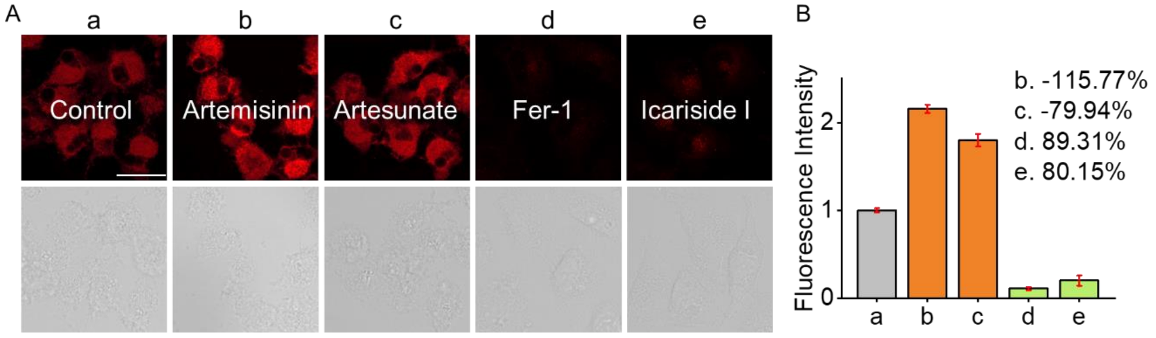

Fig. S24 (A) Fluorescence imaging of HT-1080 cells treated with 2 µM RSL3 and 25 µM various compounds for 8 h. λ_{ex} = 510 nm, λ_{em} = 600–700 nm. Scale bar, 30 µm. (B) Relative pixel intensity of images a-e in panel A. Note: the pixel intensity of image a is defined as 1.0; the results are presented as mean ± standard deviation (n = 3).

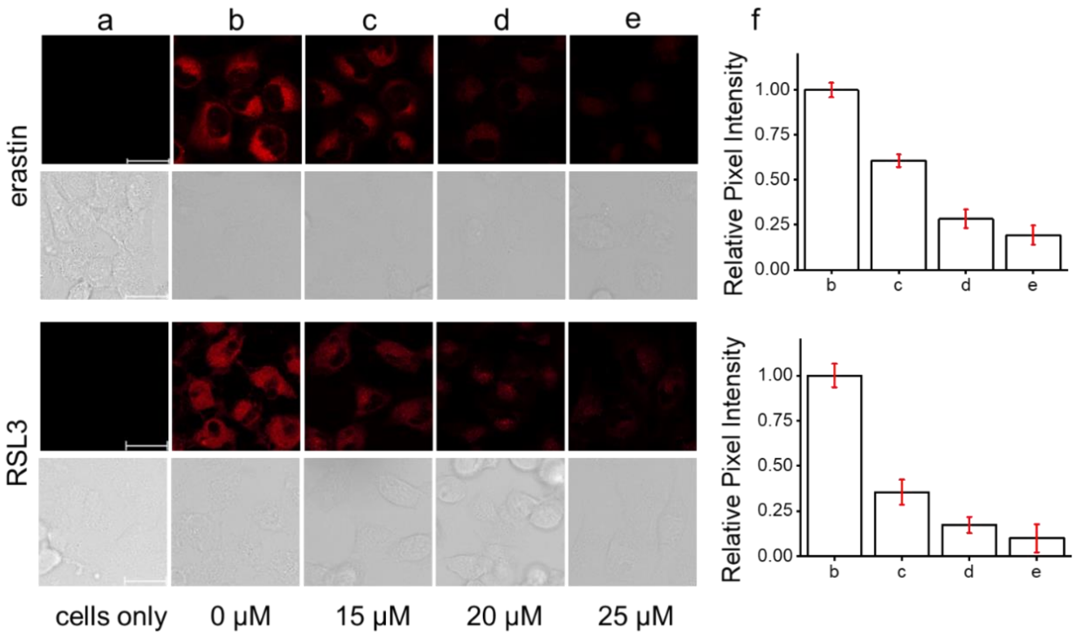

Fig. S25 Fluorescence imaging of HT-1080 cells treated with 10 µM erastin (or 2 µM RSL3) and different concentrations of icariside I for 8 h. λ_{ex} = 510 nm, λ_{em} = 600–700 nm. Scale bars, 30 µm. (f) Relative pixel intensity of the corresponding images b-e. Note: the pixel intensity of the corresponding image b is defined as 1.0; the results are presented as mean ± standard deviation (n = 3).

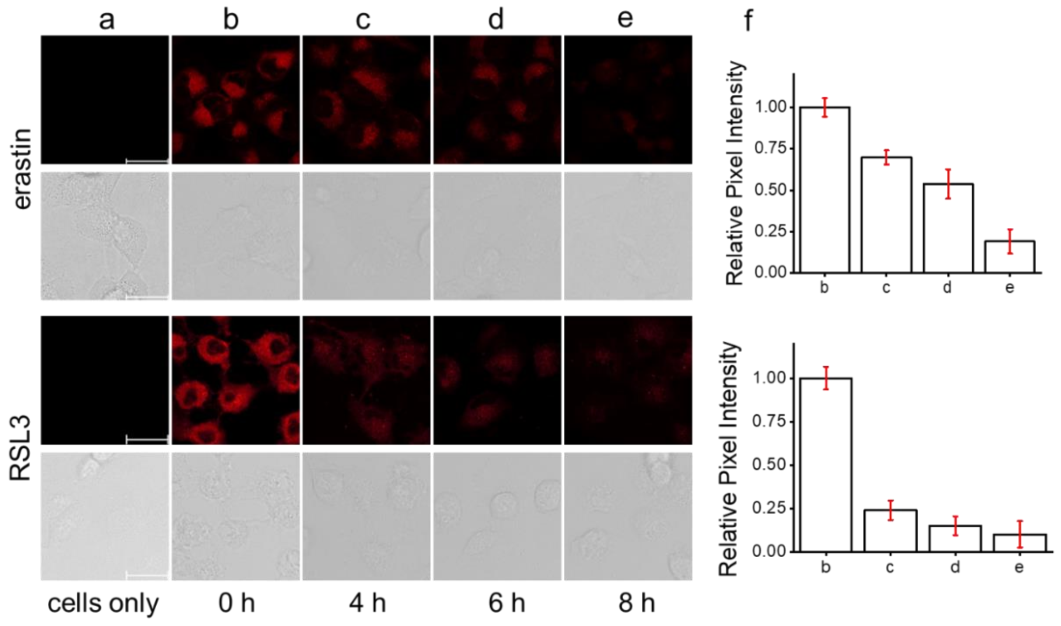

Fig. S26 Fluorescence imaging of HT-1080 cells treated with 10 µM erastin (or 2 µM RSL3) for 8 h, and then incubated with 25 µM icariside I for indicated time. λ_{ex} = 510 nm, λ_{em} = 600–700 nm. Scale bars, 30 µm. (f) Relative pixel intensity of the corresponding images b-e. Note: the pixel intensity of the corresponding image b is defined as 1.0; the results are presented as mean ± standard deviation (n = 3).

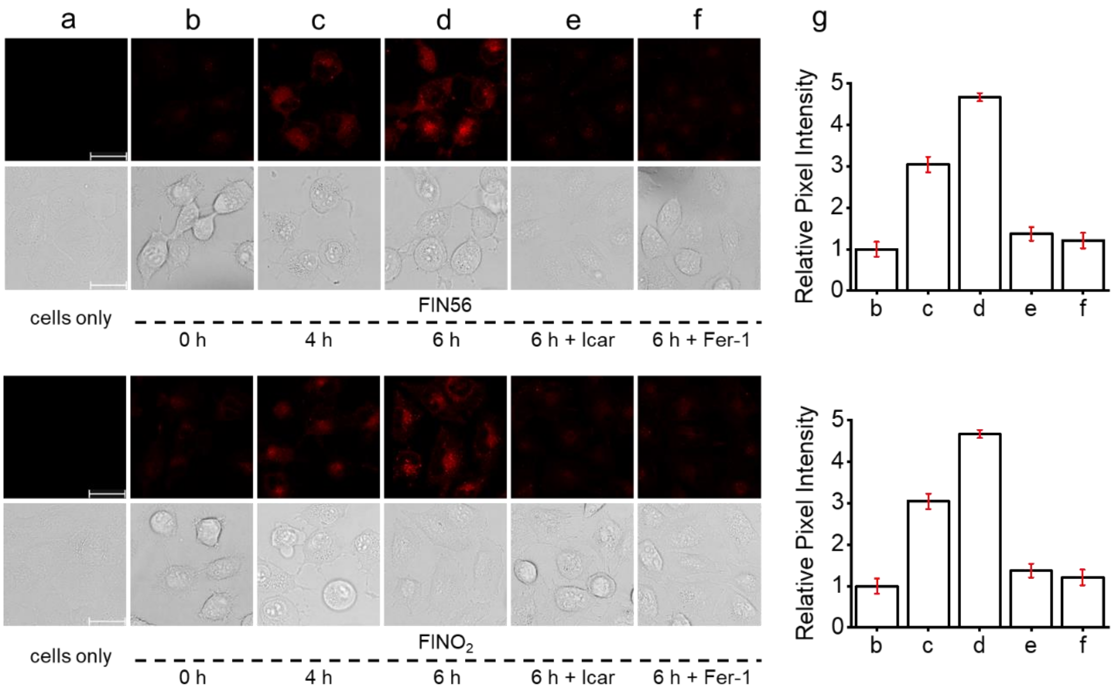

Fig. S27 Fluorescence imaging of ER •OH in HT-1080 cells during ferroptosis induced by FIN56 or $FINO_2$. (a) Cells only. (b-d) Cells pretreated with 10 μM FIN56 or 10 μM $FINO_2$ for (b) 0 h, (c) 4 h, (d) 6 h, and then incubated with 10 μM ER-OH for 30 min. (e, f) Cells pretreated with 10 μM FIN56 or 10 μM $FINO_2$ for 6 h in the presence of (e) 25 μM Icar or (f) 25 μM Fer-1, and then treated with 10 μM ER-OH for 30 min. (g) Relative fluorescence intensities of images b-f (fluorescence intensity of image b is defined as 1.0). λ_{ex} = 510 nm, λ_{em} = 600 nm-700 nm. Scar bars, 30 μm.

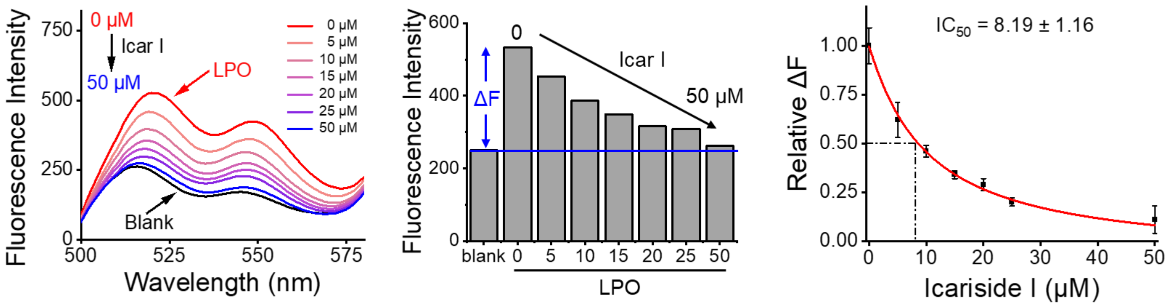

Fig. S28. Detection of lipid peroxides (LPO, 200 μM arachidonic acid + 10 μg/mL lipoxygenase) by 10 μM C11-BODIPY 581/591 in the presence of varied icariside I (Icar I). λ_{ex} = 480 nm. ΔF represents the difference in fluorescence intensity (λ_{em} = 520 nm) of C11-BODIPY 581/591 after and before the reaction with LPO.

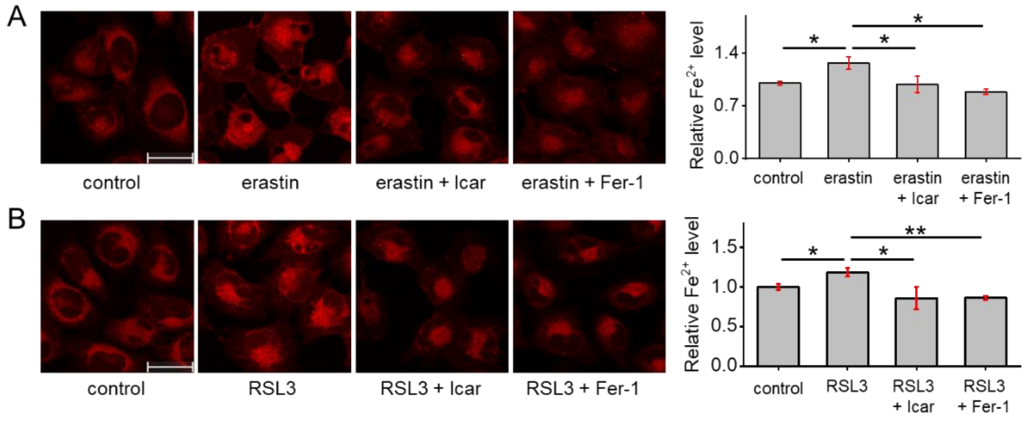

Fig. S29 Imaging of intracellular Fe^{2+} level by 10 μM RhoNox-1 in HT-1080 cells treated with different reagents for 6 h: 10 μM erastin (A); 2 μM RSL3 (B). Concentrations: 25 μM Icar, 10 μM Fer-1. λ_{ex} = 550 nm, λ_{em} = 580-700 nm. Scale bars, 30 μm. Significant differences were performed by Student's *t*-test (**p* < 0.05; ***p* < 0.01).

Fluorescence imaging of mice

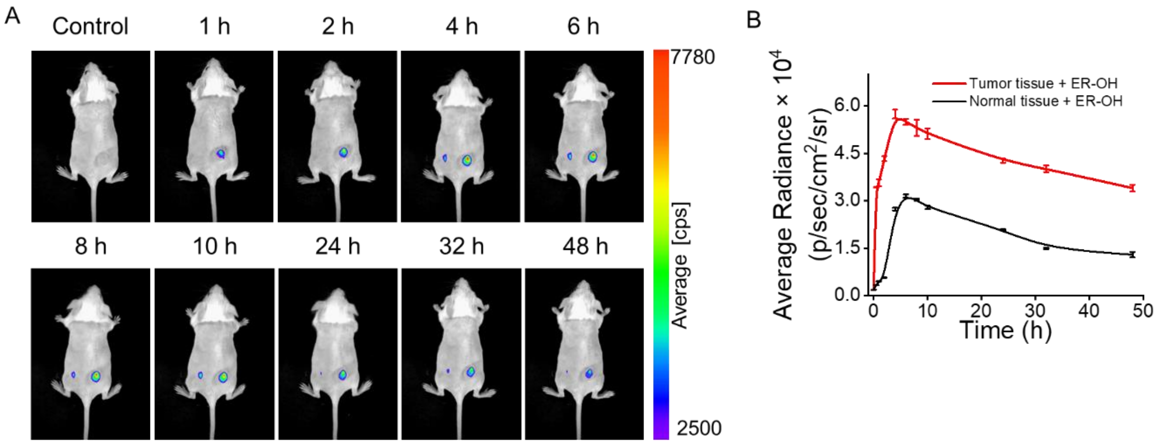

Fig. S30. *In vivo* fluorescence imaging of •OH in mice with ectopic 4T1 tumor. (A) Fluorescence imaging of the normal (left) and tumor (right) tissues after *in situ* injection with 20 μL of ER-OH (1 mM in pH 7.4 PBS, containing 10% DMF and 1 % Tween 80) at different post-injection time points. (B) Fluorescence intensities in the tumor and normal tissues (n=3).

Comparison of ER-OH with existing •OH probes

Table S1 Comparison of the analytical properties of ER-OH with some existing •OH probes.

	Structure	Analytical wavelength	Detection limit	Organelle-targeting	Applications	Ref.
1	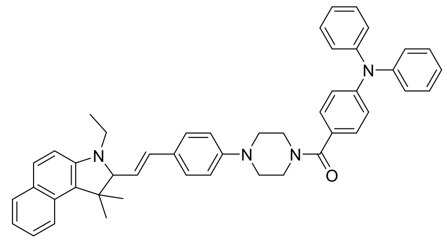	637 nm	0.26 μM	not mentioned	monitoring of ferroptosis	3
2	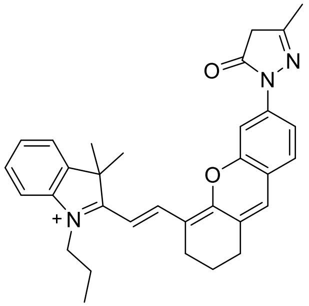	708 nm	24 nM	not mentioned	monitoring of ferroptosis	4
3	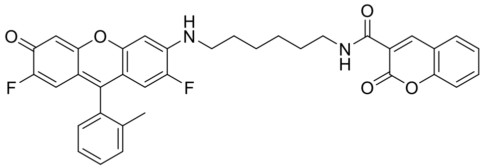	446 nm	7 μM	endoplasmic reticulum	imaging of PMA-treated cells	5

4	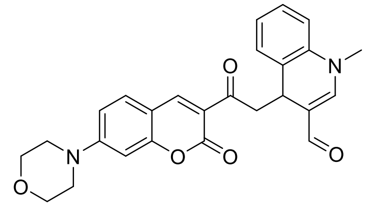	655 nm	8.9 nM	lysosome	monitoring of ferroptosis and cuproptosis;<br>imaging of tumor model	6
5	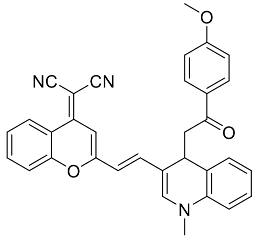	635 nm	5.77 nM	not mentioned	imaging of diabetes model	7
6	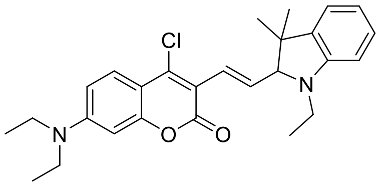	650 nm	330 nM	not mentioned	monitoring of ferroptosis	8
7	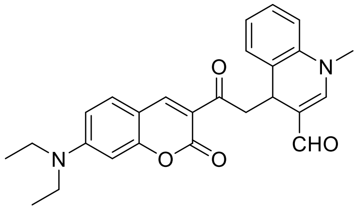	665 nm	7.7 nM	not mentioned	monitoring of ferroptosis and cuproptosis;<br>imaging of tumor model	9
8	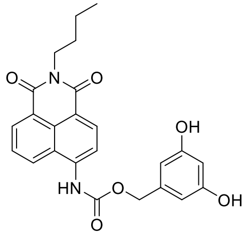	550 nm	70 nM	not mentioned	monitoring of ferroptosis;<br>imaging of AD model	10

9	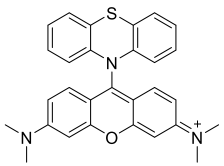	578 nm	13.6 nM	mitochondria; nucleoli	monitoring of ferroptosis	11
10	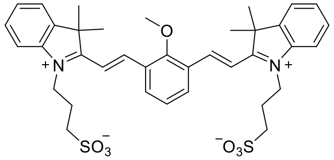	652 nm	8.6 nM	not mentioned	monitoring of ferroptosis	12
11	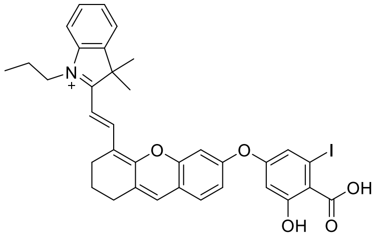	720 nm	5.30 nM	mitochondria	high-throughput screening of natural products for acute kidney injury	13
12	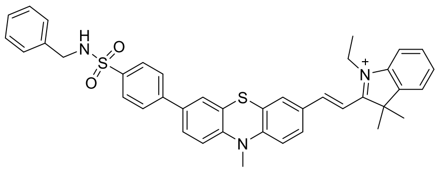	608 nm	69.65 nM	not mentioned	monitoring of ferroptosis; imaging of zebrafish; imaging of bacterial invasion model of mice	14
13	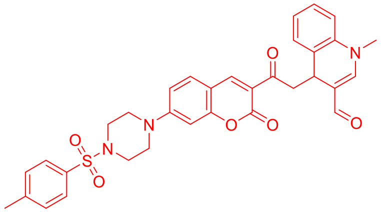	645 nm	125 nM	endoplasmic reticulum	monitoring of ferroptosis; high-throughput screening of natural ferroptosis inhibitors	This work

References

1. Y. Wu, W. Huang, D. Peng, X. A. Huang, J. Gu, S. Wu, T. Deng and F. Liu, *Org. Lett.*, 2021, **23**, 135-139.
2. P. Kumar, A. Nagarajan and P. D. Uchil, *Cold Spring Harbor Protocols*, 2018, **2018**, pdb.prot095505.
3. Z. Kang, Y. Zhou, W. Wang, S. Wang, S. Wen, H. Yu, X. Liu, J. Wang and M.-S. Yuan, *Sens. Actuators, B: Chem.*, 2025, **426**, 137121.
4. Q. Wang, H. Ji, Y. Hao, D. Jia, H. Ma, C. Song, H. Qi, Z. Li and C. Zhang, *Anal. Chem.*, 2024, **96**, 20189-20196.
5. Y. Zhao, H. Li, Z. Chai, W. Shi, X. Li and H. Ma, *Chem. Commun.*, 2020, **56**, 6344-6347.
6. X. Luo, Q. Rao, S. Wei, J. Lv, Y. Wu, M. Yang, J. Luo, J. Gao, X. Li, Z. Yuan and H. Li, *Sens. Actuators, B: Chem.*, 2025, **424**, 136951.
7. Y. Chen, X. Ji, L. Tao, C. Ma, J. Nie, C. Lu, G. Yang, E. Wang, H. Liu, F. Wang and J. Ren, *Biosens. Bioelectron.*, 2024, **246**, 115868.
8. Y. Chen, Z. Hu, M. Yang, J. Gao, J. Luo, H. Li and Z. Yuan, *Sens. Actuators, B: Chem.*, 2022, **362**, 131742.
9. H. Li, Y. An, X. Luo, J. Gao, M. Yang, X. Li, X. Li, W. Shi, Z. Yuan and H. Ma, *Chem. Eng. J.*, 2023, **476**, 146749.
10. X. Xie, J. Bian, Y. Song, G. Liu, Y. Zhao, J. Zhang, Y. Li, X. Jiao, X. Wang and B. Tang, *Anal. Chem.*, 2023, **95**, 9872-9880.
11. L.-L. Wang, Y.-Z. Mai, M.-H. Zheng, X. Wu and J.-Y. Jin, *Sens. Actuators, B: Chem.*, 2022, **373**, 132707.
12. H. Li, W. Shi, X. Li, Y. Hu, Y. Fang and H. Ma, *J. Am. Chem. Soc.*, 2019, **141**, 18301-18307.
13. H. Gao, L. Sun, J. Li, Q. Zhou, H. Xu, X.-N. Ma, R. Li, B.-Y. Yu and J. Tian, *Adv. Sci.*, 2023, **10**, 2303926.
14. Z. Kang, J. Jiang, Q. Tu, S. Liu, Y. Zhang, D.-E. Wang, J. Wang and M.-S. Yuan, *J. Am. Chem. Soc.*, 2022, **145**, 507-515.
